# Novel malaria antigen *Plasmodium yoelii* E140 induces antibody-mediated sterile protection in mice against malaria challenge

**DOI:** 10.1371/journal.pone.0232234

**Published:** 2020-05-14

**Authors:** Emily C. Smith, Keith J. Limbach, Nonenipha Rangel, Kyosuke Oda, Jessica S. Bolton, Mengyan Du, Kalpana Gowda, Jianyang Wang, J. Kathleen Moch, Sharvari Sonawane, Rachel Velasco, Arnel Belmonte, Rebecca Danner, Joanne M. Lumsden, Noelle B. Patterson, Martha Sedegah, Michael R. Hollingdale, Thomas L. Richie, John B. Sacci, Eileen D. Villasante, Joao C. Aguiar

**Affiliations:** 1 Malaria Department, Naval Medical Research Center, Silver Spring, Maryland, United States of America; 2 Henry M. Jackson Foundation for the Advancement of Military Medicine, Inc. (HJF), Bethesda, Maryland, United States of America; 3 CAMRIS International, Bethesda, Maryland, United States of America; 4 Malaria Biologics Branch, Walter Reed Army Institute of Research, Silver Spring, Maryland, United States of America; 5 Department of Microbiology and Immunology, University of Maryland School of Medicine, Baltimore, Maryland, United States of America; Ehime Daigaku, JAPAN

## Abstract

Only a small fraction of the antigens expressed by malaria parasites have been evaluated as vaccine candidates. A successful malaria subunit vaccine will likely require multiple antigenic targets to achieve broad protection with high protective efficacy. Here we describe protective efficacy of a novel antigen, *Plasmodium yoelii* (Py) E140 (PyE140), evaluated against *P*. *yoelii* challenge of mice. Vaccines targeting PyE140 reproducibly induced up to 100% sterile protection in both inbred and outbred murine challenge models. Although PyE140 immunization induced high frequency and multifunctional CD8^+^ T cell responses, as well as CD4^+^ T cell responses, protection was mediated by PyE140 antibodies acting against blood stage parasites. Protection in mice was long-lasting with up to 100% sterile protection at twelve weeks post-immunization and durable high titer anti-PyE140 antibodies. The E140 antigen is expressed in all *Plasmodium* species, is highly conserved in both *P*. *falciparum* lab-adapted strains and endemic circulating parasites, and is thus a promising lead vaccine candidate for future evaluation against human malaria parasite species.

## Introduction

An ideal *Plasmodium falciparum* malaria vaccine for non-immune travelers and individuals living in malaria endemic areas should have a protective efficacy of at least 75–80%, durability for at least six months, an acceptable safety profile, and compatibility with existing preventive measures [1, 2]. To achieve this, a vaccine must induce broad protective immune responses that target all circulating *P*. *falciparum* strains.

Development of a malaria vaccine is challenging in part due to the parasite life-cycle, with variable antigen expression and immune accessibility during parasite development in the host. Sporozoites are delivered by infected mosquitoes from the salivary glands and deposited into the skin and vasculature of the host [3], from where they transit to the blood and subsequently to the host liver. Following asymptomatic liver stage development, merozoites are released from infected hepatocytes, invade erythrocytes and initiate blood stage infection, which after several cycles of replication leads to clinical disease, as well as transmission to the invertebrate host. Vaccines designed to prevent malarial illness should target either the pre-erythrocytic stages to thwart infection, or erythrocytic stages to limit parasite density in the blood and prevent or reduce clinical symptoms.

A malaria vaccine is feasible, as immunization with *P*. *falciparum* radiation-attenuated sporozoites (RAS) by mosquito bite protected up to 100% of subjects against controlled human malaria infection (CHMI) [4, 5]. The most advanced malaria vaccine candidate, RTS,S, contains the repeat and C-terminal regions of the *P*. *falciparum* circumsporozoite protein (CSP) fused to a hepatitis B surface antigen particle. In a phase 3 clinical trial in Africa, RTS,S elicited modest protection against clinical malaria that correlated with CSP antibody and CD4^+^ T cell responses [6–8], suggesting that antibodies to sporozoites can mediate protection against malaria. RTS,S has recently been approved for pilot implementation in Ghana, Malawi, and Kenya to evaluate safety and protection from severe malaria in children. A second pre-erythrocytic vaccine, PfSPZ Vaccine, based on purified, cryopreserved, radiation-attenuated sporozoites, induced 100% sterile protection in humans against CHMI using the immunizing *P*. *falciparum* strain [9], and durable (six months) protection against clinical malaria in African adults after a further boost [10]. However, high level protection against multiple field strains has yet to be demonstrated [9, 11, 12]. RAS-induced protection is thought to be mediated by cytotoxic T lymphocytes recognizing infected hepatocytes, but antibodies to sporozoites likely contribute as well [13, 14]. The liver stage targets are CSP, as well as largely unidentified pre-erythrocytic antigens [9]. To date, malaria vaccine development has focused on a tiny fraction of the approximately 2,000 pre-erythrocytic proteins presumed to be involved in the RAS-induced human immune response and protection [15, 16].

Malaria vaccines targeting erythrocytic stage antigens, such as merozoite surface protein 1 (MSP1), apical membrane antigen 1 (AMA1), glutamate-rich protein (GLURP)/merozoite surface protein 3 (MSP3), Combo B [MSP1, merozoite surface protein 2 (MSP2), and ring-infected erythrocyte surface antigen (RESA)], and reticulocyte-binding protein homolog 5 (RH5), have been evaluated in clinical trials. Some of these vaccines elicited allele-specific immunity or reduced parasite density [17]. However, in general, none of these vaccines were broadly protective.

Due to the high protective efficacy of RAS vaccines and the need to increase protective immunity induced by RTS,S, we previously sought to identify novel pre-erythrocytic antigen targets of the PfRAS immune response. We screened antigens selected by bioinformatics and expression database analyses using PfRAS immune sera and peripheral blood mononuclear cells [18]. A similar analysis of *P*. *yoelii* pre-erythrocytic antigens led to the identification of a two-antigen vaccine that consistently elicited higher levels of sterile protection than a comparable PyCSP vaccine in the *P*. *yoelii* mouse model [19]. More recently, we have further evaluated additional orthologous *P*. *yoelii* pre-erythrocytic antigens for sporozoite expression and localization, recall T cell reactivity from splenocytes of PyRAS-immunized mice, and the capacity to induce sterile protection in outbred CD1 mice against a *P*. *yoelii* sporozoite challenge. Here, we report the identity of a novel antigen, PyE140, which induced up to 100% protection against sporozoite and infected red blood cell challenges in outbred CD1 and inbred BALB/c mice which persists for at least twelve weeks. We show that PyE140 is expressed in sporozoites, liver, and blood stages, and is a target of protective antibody responses directed against blood stage parasites. PyE140 shares 35% amino acid sequence identity with PfE140, which is highly conserved among *P*. *falciparum* strains. These data demonstrate that PfE140 is a promising human malaria vaccine candidate with the potential to elicit blood stage protection against diverse *P*. *falciparum* circulating parasites.

## Materials and methods

### Re-annotation of the PyE140 gene

Alignment of the amino acid sequences of the *P*. *yoelii* 17XNL (PY06306), *P*. *berghei* ANKA (PBANKA_020900) and *P*. *falciparum* 3D7 (PF3D7_0104100; PFA0205w) E140 orthologues from the PlasmoDB database [20] indicated that the *P*. *berghei* and *P*. *falciparum* orthologues, but not the *P*. *yoelii* orthologue, had substantial homology throughout the protein. Specifically, PlasmoDB predicted that the PyE140 protein was 479 amino acids long and was encoded by an exon 1 of 1,362 bp and an exon 2 of 78 bp. By contrast, PlasmoDB predicted that the PfE140 protein was 791 amino acids long and was encoded by an exon 1 of 775 bp and an exon 2 of 1,601 bp and the PbE140 protein was 818 amino acids long and was encoded by an exon 1 of 862 bp and an exon 2 of 1,595 bp. These results suggested that the PlasmoDB annotation of the PyE140 orthologue (PY06306) was not correct. A re-annotated PyE140 gene was created by examining the *P*. *yoelii* genomic sequence containing the incorrectly annotated PY06306 gene from the PlasmoDB database with the nucleotide sequences of the PbE140 and PfE140 genes. The re-annotated PyE140 gene is 2,451 bp, with an exon 1 of 856 bp and an exon 2 of 1,595 bp, and encodes a protein that is 816 amino acids long (S1 Fig). When aligned, the re-annotated PyE140 protein and the PbE140 protein are 86% homologous, whereas the incorrectly annotated PY06306 protein and the PbE140 protein are 46% homologous. Subsequent to the above analysis, the genomes for *P*. *yoelii* strains 17X and YM became available in PlasmoDB. The PyE140 genes in these strains, PY17X_0210400 and PYYM_0211900, respectively, are 100% identical to the re-annotated PyE140 17XNL sequence.

### Generation of DNA and HuAd5 vaccine vectors

Exon 1 and exon 2 of the re-annotated PyE140 gene were each cloned into pCR2.1-TOPO (ThermoFisher Scientific, Inc., Waltham, MA). Exon 1 was generated by PCR with *P*. *yoelii* genomic DNA and the primers, E140P17 (5’-GGATCCACCATGGGAGACGTTGACAATGTG-3’) and E140P19 (5’-GCTAGCATGTTGAATCCGGTTTGGTACTTTAAAACAATAGGCAAGCCCTTATTC-3’). E140P19 contains sequences from the junction of exon 1 and exon 2, and includes a *Nhe*I site located in exon 2. Exon 2 was generated by PCR with *P*. *yoelii* genomic DNA and the primers, E140P20 (5’-GTACCAAACCGGATTCAACATG-3’) and E140P21 (5’-GGATCCTCAAATAATCTTCATTTGATGG-3’). Primers were purchased from Eurofins Genomics, LLC (Louisville, KY). Exon 1 was then cloned into a DNA vaccine vector, VR1020 (Vical, San Diego, CA), downstream from a human cytomegalovirus (HCMV) immediate early (IE) promoter and in-frame with a human tissue plasminogen activator (TPA) signal sequence. Utilizing the *Nhe*I site, exon 2 was then cloned into the DNA vaccine vector containing exon 1, resulting in a DNA vaccine vector containing a full-length PyE140 gene, DNA-PyE140na (native sequence). Due to a PCR-generated error, this construct contains a 1 bp substitution that changes the serine at position 537 to a glycine. The full-length PyE140 gene from this vector was then cloned into a HuAd5 shuttle plasmid, downstream from a HCMV IE promoter. This plasmid was then used to generate an E1-, partial E3-, E4-deleted, replication-incompetent HuAd5 vector, HuAd5-PyE140na, in 293-ORF6 cells [21], using a plasmid-based construction system [22] that positions the HCMV IE-PyE140na expression cassette into the E1 region of the HuAd5 genome.

The synthetic PyE140 gene, codon-optimized for optimal expression in mice, was generated by GenScript Biotech (Piscataway, NJ). This codon-optimized PyE140 gene, PyE140co, was cloned into a HuAd5 shuttle plasmid, downstream from a HCMV IE promoter, which was used to generate an E1-, partial E3-, E4-deleted, replication-incompetent HuAd5 vector, HuAd5-PyE140co. HuAd5-PyE140na was used for all immunizations, except for those described in Figs 6 and 8, and S9 Fig, where HuAd5-PyE140co is specified. (This virus does not contain the above substitution and encodes serine at position 537.).

The E1-, E3-deleted, replication incompetent HuAd5 vector, HuAd5-PfE140, was generated by OD260, Inc. (Boise, ID) and contains a synthetic *P*. *falciparum* 3D7 (PF3D7_0104100; PFA0205w) E140 gene, codon-optimized for optimal expression in mice, under the control of a HCMV IE promoter and bovine growth hormone polyA signal in place of the E1 region of the HuAd5 genome.

DNA vaccines were manufactured by Puresyn, Inc. (Malvern, PA) and contained endotoxin at levels less than 0.1 EU/μg of DNA. HuAd5 vaccines were negative for the following pathogens: *Mycoplasma* spp, *M*. *pulmonis*, MHV, MVM, MPV 1-5, TMEV, Sendai, PVM, MNV, REO3, EDIM, Ectro, LCMV, Polyoma, LDV, MAV 1, MAV 2, MCMV, *Helicobacter* spp, and *C*. *piliforme* (IDEXX Laboratories, Inc., Westbrook, ME).

### Ethics statement

The study protocols were reviewed and approved by either the University of Maryland, Baltimore or the Walter Reed Army Institute of Research (WRAIR)/Naval Medical Research Center (NMRC) Institutional Animal Care and Use Committee in compliance with all applicable federal regulations governing the protection of animals and research. The experiments reported herein were conducted in an AAALACi accredited facility in compliance with the Animal Welfare Act and per the principles set forth in the "Guide for Care and Use of Laboratory Animals,” Institute of Laboratory Animals Resources, National Research Council, National Academy Press, 2011.

### Mice

Six to nine week old female CD1 (Charles River Laboratories, Wilmington, MA) or BALB/c (Charles River Laboratories) mice were used in all experiments, with the exception of the mice in Fig 8 (liver burden analysis following subcutaneous challenge), which were 11 weeks old on the date of immunization. All mice were euthanized by CO_2_ vapor inhalation followed by cervical dislocation. CO_2_ was delivered from commercial pressurized cylinders via regulated flow valve in accordance with the 2013 American Veterinary Medical Association Guidelines on Euthanasia. The procedures and manipulations did not necessitate the use of anesthesia or analgesia.

### Immunizations

All DNA and HuAd5 immunizations were given by intramuscular (IM) injection in the *tibialis anterior* muscle with a six week interval between the prime and the boost. DNA vaccines were administered at a dose of 100 μg in a volume of 100 μL (50 μL per muscle). HuAd5 vaccines were typically administered at 1 x 10^10^ particle units (pu) in a volume of 100 μL (50 μL per muscle). Doses less than 1 x 10^10^ pu were also used, as described in the legend of Fig 6. IM injections were performed using a tuberculin syringe and a 30G needle at a depth of 2 mm.

### Malaria parasites

*P*. *yoelii* (17XNL) parasites were maintained by alternating passage of the parasites in *Anopheles stephensi* mosquitoes and CD1 mice. Sporozoites were isolated from the salivary glands of infected mosquitoes by hand dissection 14–21 days after blood meal. To acquire blood stage parasites, mice were bled from the tail vein ~7 days after infection with *P*. *yoelii* sporozoites at the peak of parasitemia. Parasites were used either for the preparation of slides for immunofluorescence assay (IFA; described below), Western blot, or for challenge of mice. For preparation of blood stage IFA slides, schizonts were enriched by Percoll purification after short-term culture [23]. Liver sections were prepared as previously described [19]. Sporozoites and blood stage parasites were air dried on 12-well glass slides for IFA and stored at -80°C. For sporozoite challenge, sporozoites were resuspended in E199 media containing 5% normal mouse sera, which was harvested from mice housed at NMRC and tested negative for pathogens as described above for HuAd5. CD1 or BALB/c mice were injected intravenously in the lateral tail vein with 200 μL containing 300 or 100 sporozoites, respectively. For evaluation of liver stage parasite burden, BALB/c mice were injected subcutaneously with either 20,000 or 5,000 sporozoites in 200 μL. For blood stage challenge, infected blood was resuspended in E199 media containing 5% normal mouse sera, and CD1 or BALB/c mice were injected intravenously in the lateral tail vein with 10,000 infected red blood cells in 200 μL. Parasitemia was evaluated by examining Giemsa-stained thin blood smears. Mice were considered positive if blood stage parasites were observed in any blood smear. To identify mice that had delayed emergence of parasites after sporozoite challenge, we evaluated the days to parasitemia from all null-immunized and naïve mice (Figs 2–6). The longest time to parasitemia in these negative control mice was six days, with a mean of 4.3 days to parasitemia. (Mice were not tested before day 4 after challenge). Therefore, we defined a delay to parasitemia as day seven or later following sporozoite challenge, which exceeds five standard deviations above the mean of the negative control mice. Data were plotted using GraphPad Prism version 7.05 (GraphPad Software, Inc., San Diego, CA) in Kaplan-Meier plots with log rank (Mantel-Cox) to compare the survival curves for all mice in each group.

*P*. *falciparum* schizonts (strains 3D7, FVO, Dd2, 7G8, HB3, HHSS, SA250, NF54, and M24) were obtained from the WRAIR Genetics and Parasite Biology Laboratory. Parasites were cultured and schizonts purified as previously described [24].

### Antibodies

For T cell depletion, anti-CD4 (clone GK1.5; ATCC, Manassas, VA) and anti-CD8 (clone 2.43; ATCC) antibodies were purified from ascites (Harlan Laboratories, Indianapolis, IN). Rat IgG was purchased from Protein Mods (Madison, WI). For intracellular cytokine staining and/or assessment of T cell depletion efficiency, anti-CD14 Cy7APC (clone SA14-2), anti-CD19 Cy7APC (clone 6D5), anti-CD44 Pacific Blue (clone IM7), anti-CD3 Alexa 700 (clone 17A2), anti-CD4 Brilliant Violet 605 (clone RM4-5), anti-CD8a Brilliant Violet 510 (clone 53–6.7), anti-CD19 BV711 (clone 6D5), anti-CD8b.2 PE (clone 53–5.8) were purchased from Biolegend (San Diego, CA), anti-CD62L Brilliant Violet 786 (clone MEL-14), anti-CCR7 Brilliant Violet 650 (clone 4B12), anti-TNF Cy7PE (clone MP6-XT22), anti-IFN-γ APC (clone XMG1.2), anti-IL-2 FITC (clone JES6-5H4) were purchased from Becton Dickinson (San Jose, CA), and anti-MIP1α PE was purchased from R&D Systems (Minneapolis, MN). All antibodies above are anti-mouse. Anti-PyCSP (clone NYS1), used for passive transfer, was previously described [25].

### Western blots

#### Parasite expression of PyE140 protein

*P*. *yoelii* sporozoites were freshly hand-dissected from salivary glands of *Anopheles stephensi* mosquitoes 21 days post-infection. *P*. *yoelii* schizonts were harvested from *P*. *yoelii*-infected mouse blood with 5% parasitemia. Blood stage parasites were *in vitro* cultured for four hours until most parasites were mature schizont stages and then purified on a Percoll-alanine gradient as described previously [23]. 3.6 × 10^5^ *P*. *yoelii* sporozoites and 2 × 10^5^ parasitized or uninfected red cells were lysed in reducing Bolt LDS sample buffer (Invitrogen Corp., Carlsbad, CA), boiled, and electrophoresed on a 4–12% Bis-Tris SDS-PAGE (Invitrogen). Protein molecular weight standards are Magic Mark XP Western Protein Standard (Invitrogen). The resolved protein samples were transferred onto polyvinylidene difluoride (PVDF) membranes (Invitrogen) and processed for detection using the iBind Flex Solution Kit and the iBind Flex Western Device (Invitrogen) that includes membrane, buffers for blocking, washes, and antibody incubations. Proteins were probed with polyclonal sera induced in CD1 mice with a DNA-PyE140na-HuAd5-PyE140co prime-boost immunization regimen at a 1:500 dilution. Secondary antibody was horseradish peroxidase (HRP)-conjugated goat anti-mouse IgG Fc (polyclonal; Southern Biotech, Birmingham, AL) at a 1:1,000 dilution. After extensive washing, the blots were developed with ECL Western Blotting Substrate (Invitrogen) and chemiluminescence captured on a ChemiDoc Touch Imaging System (Bio-Rad Laboratories, Hercules, CA).

#### Cellular expression of PyE140 protein

293-ORF6 [21] cells in 60 mm dishes were infected with HuAd5 null, HuAd5-PyE140na, or HuAd5-PyE140co at a multiplicity of infection (MOI) of either 500 or 6,500 pu/cell or transfected with 8 μg of DNA-PyE140na using lipofectamine 2000CD (Invitrogen). Cell lysates were harvested in SDS protein gel loading solution (Quality Biological Inc., Gaithersburg, MD) 24 hours or 48 hours post-infection/transfection, run on a 4–20% Tris-Glycine acrylamide gel (Invitrogen) and transferred to an Immobilon-P PVDF membrane (Millipore Corp., Bedford, MA). The primary antibody was a 1:100 dilution of sera from mice immunized with DNA-PyE140na and HuAd5-PyE140na. The secondary antibody was a 1:2,500 dilution of polyclonal goat anti-mouse IgG + IgM conjugated to alkaline phosphatase (Applied Biosystems Inc., Foster City, CA). The marker is BenchMark Prestained Protein Ladder (Invitrogen). Proteins were visualized with an alkaline phosphatase Western-Light Chemiluminescent Detection System (Tropix Inc., Bedford, MA) and an alkaline phosphatase colorimetric substrate (KPL Inc., Gaithersburg, MD).

### Peptides

A PepSet^™^ library, crude purity, of 15mer peptides overlapping by 10 amino acids (Mimotopes, Victoria, Australia) spanning full length PyE140 was resuspended at a concentration of 20 mg/mL in dimethyl sulfoxide (DMSO) based on an assumed 3 mg yield per peptide and an average purity based on the plate controls of 63.25%. Individual peptides were pooled in two pools of 81 (PyE140-A, amino acids 1–415) and 80 (PyE140-B, amino acids 406–816) peptides corresponding to the N- and C-termini, respectively, at approximately 200 μg/mL per peptide in DMSO.

### T cell depletion

Mice were depleted of CD4^+^ and/or CD8^+^ T cells by intraperitoneal (IP) administration of anti-CD4 and/or anti-CD8 monoclonal antibodies. To deplete CD4^+^ T cells, IP injections of 1.0 mg/mouse of the anti-CD4 monoclonal antibody GK1.5 were given on days -5, -3, -1, and +1 relative to the date of challenge with *P*. *yoelii* sporozoites on day 0. To deplete CD8^+^ T cells, IP injections of 0.5 mg/mouse of the anti-CD8 monoclonal antibody 2.43 were given on days -5, -3, and -1 relative to the date of challenge with *P*. *yoelii* sporozoites on day 0. To deplete both CD4^+^ and CD8^+^ T cells, mice received both antibodies as described above in a single injection on days -5, -3 and -1, and the anti-CD4 monoclonal antibody on 1 day after challenge. Non-depleted mouse controls in depletion experiments received corresponding IP injections of 1.0 mg of purified rat IgG, according to the schedule for CD4^+^ T cell depletion. Antibodies were resuspended in sterile phosphate-buffered saline (PBS) to 500 μL total volume per injection.

### Passive transfer of sera

Mice were immunized by a DNA-HuAd5 PyE140na prime-boost regimen as described above and terminally bled by cardiac puncture. Sera were collected in microvolume serum separator tubes (BD Biosciences) and centrifuged at 15,000 rpm for 10 minutes. Sera from eight immunized mice were transferred to fourteen naive mice by IP injection approximately 30 hours before challenge, and again from a second set of eight immunized mice to the same fourteen naïve mice at approximately five to six hours before challenge. Recipient mice were bled at approximately three hours before challenge to evaluate the titer of transferred antibodies in the recipient mice. Immunized mice that did not serve as sera donors were challenged in parallel as positive controls. Null-immunized and naïve mice served as negative controls. An additional group of mice received 500 μg of PyCSP monoclonal antibody, NYS1 [26].

### Cellular staining and flow cytometry

Spleens and livers from immunized mice (n = 6 per group) were harvested two weeks after the final boost. Livers were perfused and intrahepatic lymphocytes were harvested as described [27]. Splenocytes and intrahepatic lymphocytes were stimulated with either PyE140-A or PyE140-B peptide pools at 2 μg/mL in 1% DMSO, or anti-CD3/anti-CD28 beads (Miltenyi Biotec, Auburn, CA) at a concentration of 8 million beads per mL. All stimulations were performed in complete RPMI [RPMI 1640 containing 25 mM HEPES, with 1% MEM NEAA, 1% sodium pyruvate, 1X Penicillin/Streptomycin, 2 mM glutamine (Invitrogen), 0.05 mM ß-mercaptoethanol (Sigma Aldrich, St. Louis, MO), and 10% fetal calf serum (Sigma Aldrich)] with 20 μg/mL brefeldin A (Sigma Aldrich). Splenocytes were stimulated for 4 hours at 37°C in a 96-well v-bottom plate and then transferred to 4°C overnight (<18 hours). Cells were washed twice with 1X PBS (Invitrogen) and then stained with a titrated quantity of LIVE/DEAD^®^ Fixable Blue Dead Cell Stain for UV excitation (Invitrogen) and incubated in the dark at room temperature for 20 minutes. Cells were washed twice with FACS wash buffer [1X PBS with 1% bovine serum albumin (EMD Chemicals, Philadelphia, PA) and 0.1% sodium azide (Sigma Aldrich)]. Cells were then surface stained with titrated quantities of anti-mouse antibodies (anti-CD14 Cy7APC, anti-CD19 Cy7APC, anti-CD44 Pacific Blue, anti-CD62L Brilliant Violet 786, and anti-CCR7 Brilliant Violet 650) in FACS wash buffer for 20 minutes at room temperature in the dark. Cells were washed twice with FACS wash buffer, and then incubated with 100 μL of cytofix/cytoperm (BD Biosciences) for 20 minutes at 4°C in the dark. Cells were washed twice with 1X cytofix/cytoperm wash buffer (BD Biosciences) and then stained with anti-mouse intracellular antibodies (anti-CD3 Alexa 700, anti-CD4 Brilliant Violet 605, anti-CD8a Brilliant Violet 510, anti-TNF Cy7PE, anti-IFN-γ APC, anti-IL-2 FITC, and anti-MIP1α PE) in 1X cytofix/cytoperm wash buffer for 30 minutes at room temperature in the dark. Cells were washed three times in 1X cytofix/cytoperm wash buffer and then resuspended in 1% formaldehyde (Tousimis, Rockville, MD) in 1X PBS. Samples were acquired by a LSRII cytometer with the high throughput sampler (BD Biosciences). Data were analyzed by FlowJo version 9.4.11 (Tree Star, Inc., Ashland, OR) and by SPICE version 5.35 (downloaded from http://exon.niaid.nih.gov) [28].

To evaluate CD4^+^ and CD8^+^ T cell depletion from spleens of immunized mice, cryopreserved splenocytes were thawed and stained as above using anti-mouse antibodies for surface (anti-CD14 Cy7APC, anti-CD19 BV711, anti-CD3 ALX700, anti-CD4 BV605, and anti-CD8b.2 PE) and/or intracellular (anti-CD3 ALX700, anti-CD4 BV605, and anti-CD8b.2 PE) staining. Samples were acquired on a BD Fortessa cytometer (BD Biosciences) in tubes. Data were analyzed using FlowJo version 9.9.5 (Tree Star, Inc.).

### Immunofluorescence assay (IFA)

*P*. *yoelii* or *P*. *falciparum* sporozoites or schizonts were air dried on 12-well glass slides and frozen at -80°C. Liver tissue sections containing *P*. *yoelii* parasites were cut on a cryostat and stored at -80°C. Serial dilutions of mouse sera in 1X PBS with 2% bovine serum albumin (Sigma Aldrich) were incubated on the slides for 1 hour at 37°C in a humidity chamber containing damp paper towels. The slides were washed four times with 1X PBS for five minutes. Goat anti-mouse IgG FITC (polyclonal, KPL, Milford, MA) was added in 0.0005% Evans blue in water obtained from a Milli-Q Synthesis (96 Range) water purification system (Millipore Corp.), and the slides were incubated for 1 hour at 37°C in the humid tray and then washed four times with 1X PBS for five minutes. Slides were mounted with coverslips using VECTASHIELD^®^ HardSet^™^ Antifade Mounting Medium with 4′,6-diamidino-2-phenylindole (DAPI; Vector Laboratories, Burlingame, CA). Slides were imaged on a fluorescent microscope equipped with a digital camera (Olympus, Tokyo, Japan).

The end-point IFA titer was defined as the highest antibody dilution that showed positive specific fluorescence [15]. Data were plotted using GraphPad Prism version 7.05 (GraphPad Software, Inc.) by box and whisker plots. The boxes extend from the 25^th^ to 75^th^ percentile, the whiskers extend from the minimum to maximum values, and the horizontal line indicates the median value. Statistical analysis was performed by Mann-Whitney.

### Liver stage burden analysis by RT-qPCR

At 42 hours after sporozoite challenge, whole livers were harvested from mice, finely minced using surgical scissors, and stored in 10 mL RNA Later (Invitrogen) at 4°C for a minimum of 16 hours to a maximum of 1 week. Preserved liver tissue was strained using 70 μm cell strainers and the tissue was transferred to 15 mL TRIzol (Invitrogen). The full quantity of each individual mouse liver was homogenized using an Omni Tissue Homogenizer (OMNI International, Inc., Kennesaw, GA) with hard tissue probes. Liver homogenate was stored at -80°C in 1 mL aliquots. RNA isolation was performed from 400 μL liver homogenate, after removing debris by centrifugation, using TRIzol according to the manufacturer’s specifications with 50% ethanol rather than 70%. Among 48 samples, the mean eluted RNA concentration was 504 ng/μL (range 350 ng/μL to 770 ng/μL) and the mean OD 260/280 ratio was 2.01 (range 1.99 to 2.06) as determined by a NanoDrop ND1000 Spectrophotometer (ThermoFisher Scientific, Inc.). DNase I treatment was performed on 2.5 μg RNA for each sample (Invitrogen) for 15 minutes at room temperature in a 25 μL total reaction volume. DNase I was inactivated by adding 2.5 μL 25 mM EDTA and incubating at 65°C for 10 minutes. Reverse transcription was performed using 10 μL (0.91 μg) RNA using the High Capacity cDNA Reverse Transcription kit (ThermoFisher Scientific, Inc.) with random primers and 4 mM dNTPs. A control with no reverse transcriptase was performed for each sample to ensure the lack of contaminating DNA. Reverse transcription was performed in a T100 thermal cycler (Bio-Rad Laboratories) at 25°C for 10 minutes, 37°C for 120 minutes, and 85°C for 5 seconds, followed by a 4°C hold. The resulting cDNA was stored overnight at -30°C. Quantitative PCR was performed using the Taqman Universal PCR Master Mix (ThermoFisher Scientific, Inc.) with 5 μL cDNA, 0.3 μM forward and reverse primers, 0.2 μM probe and 1X Taqman buffer for amplification of Py18S, and using 0.1 μM forward and reverse primers with 0.2 μM probe for amplification of murine *Gapdh*. Reactions were performed in duplicate for each target in a final volume of 25 μL using DEPC water (Quality Biological, Inc.). Quantitative PCR was performed on an ABI 7500 Fast Real-Time PCR System (ThermoFisher Scientific, Inc.) with the following method: 50°C for 2 minutes, 95°C for 10 minutes, followed by 45 cycles of 95°C for 15 minutes and 60°C for 1 minute. The Py18S forward primer was 5'-CTTGGCTCCGCCTCGATAT-3', the Py18S reverse primer was 5'-TCAAAGTAACGAGAGCCCAATG-3', and the probe sequence was [6~FAM]CTGGCCCTTTGAGAGCCCACTGATT[TAMRA~Q], all purchased from Eurofins Genomics LLC [29]. Murine *Gapdh* was amplified using the Taqman Rodent GAPDH Control Reagents Kit (ThermoFisher Scientific, Inc.). Copy number was determined using a single diluted plasmid standard encoding both the Py18S and murine *Gapdh* target sequences in a single construct.

To generate the above plasmid standard, a plasmid encoding the Py18S sequence in the PCR-Script Amp backbone [29] was digested with *Mfe* I (New England BioLabs, Ipswich, MA) to generate a 948 bp fragment of Py18S from positions 549 to 1497. A plasmid encoding murine *Gapdh* (clone ID 6493407, Open Biosystems, Huntsville, AL) was linearized with *Mfe* I. Following agarose gel electrophoresis, the products were gel purified (Qiagen, Germantown, MD). Following subsequent ethanol precipitation, the Py18S insert was ligated to the linearized *Gapdh* plasmid using T4 DNA ligase (New England BioLabs). Clones were screened by PCR using the *Gapdh* forward primer and the Py18S reverse primer to select clones with both sequences in the same orientation. Plasmid standards were prepared by maxi prep (Qiagen). Serial dilutions were prepared in DEPC water (Quality Biological, Inc.) containing 30ng/μL yeast tRNA (Invitrogen).

## Results

### Re-annotation of the PyE140 gene

Alignment of the *P*. *yoelii*, *P*. *berghei*, and *P*. *falciparum* E140 gene sequences from PlasmoDB [20] indicated that the PyE140 gene was not annotated correctly. Using these sequences, the PyE140 gene was identified by re-annotating sequences from the hypothetical PY06305 and PY06306 genes in PlasmoDB into a single gene encoding 816 amino acids. The re-annotated PyE140 DNA and amino acid sequences and alignment to PfE140 are shown in S1 Fig. The PfE140 gene is identified in PlasmoDB as a conserved membrane protein with unknown function.

### E140 antigen has orthologous sequences in all known *Plasmodium* species

**Fig 1:**
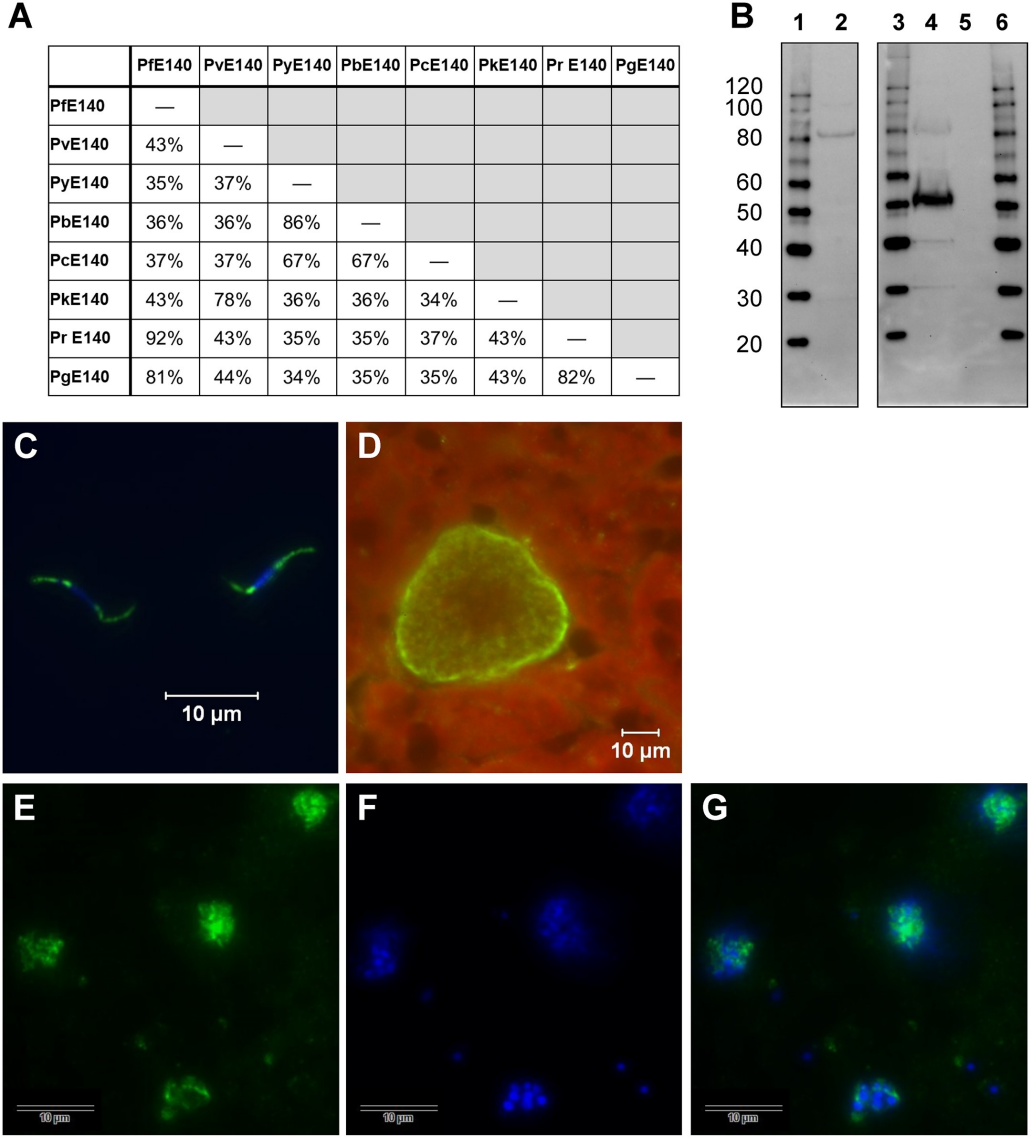
PyE140 ortholog identity and expression in sporozoite, liver, and blood stage parasites. (A) Sequence identity among *Plasmodium* species was determined by aligning E140 amino acid sequences as listed in the Plasmodium 100 Genomes initiative, Broad Institute, broadinstitute.org by BLAST [31]. Pf–*P*. *falciparum* 3D7, Pv–*P*. *vivax* Sal-1, Py–*P*. *yoelii* 17XNL, Pb–*P*. *berghei* ANKA, Pc–*P*. *chabaudi* chabaudi, Pk–*P*. *knowlesi* H, Pr–*P*. *reichenowi* G01, Pg–*P*. *gaboni* SY75. (B) Western blot analysis of *P*. *yoelii* salivary gland sporozoites (lane 2), mature schizonts (lane 4), and uninfected mouse red blood cells (lane 5). Parasite lysate was obtained from 3.6 × 10^5^ *P*. *yoelii* sporozoites and 2 × 10^5^ parasitized and non-infected red blood cells, respectively. Protein molecular weight standard (MagicMark XP, ThermoFisher Scientific, Inc.) is shown in lanes 1, 3, and 6 and the sizes in kDa are listed to the left for reference. The blot was probed with polyclonal sera from mice immunized against PyE140 at a 1:500 dilution. The serum detects predominant bands for PyE140 at ~82 kDa for sporozoites and a smaller protein (~53 kDa) for schizonts. The lanes shown are derived from a single blot with lanes containing duplicate and additional size markers excluded. (C-G) Sera from mice immunized against PyE140 were used to detect PyE140 expression in different stages of malaria parasites. Green fluorescence indicates PyE140. (C) Sporozoite IFA: PyE140 is expressed in discrete patches at both the apical and posterior ends of the sporozoite. Scale bar indicates 10 μm. Merged image also shows DAPI nuclear counterstain (blue). (D) Liver stage IFA: PyE140 is largely expressed in the periphery of 42 hour *P*. *yoelii* liver stage parasites and more weakly internally near developing liver stage merozoites. Scale bar indicates 10 μm. (E-G) Blood stage IFA—PyE140 is expressed in individual merozoites in late stage schizonts: (E) PyE140 (green), (F) DAPI nuclear counterstain (blue), (G) merged image shows PyE140 (green) and DAPI nuclear counterstain (blue). Scale bar indicates 10 μm.

E140 amino acid sequences from different *Plasmodium* species are highly homologous (Fig 1A), especially between PfE140 and the *P*. *falciparum*-related species, *P*. *reichenowi* (92%) and *P*. *gaboni* (81%), consistent with the sequence conservation observed in other genes from these related species [30]. We also observed a high degree of conservation between rodent parasites, such as Py-Pb (86%), Py-Pc (67%), and Pb-Pc (67%). Both PfE140 and PyE140 proteins contain five predicted transmembrane domains (S2 Fig), and are thus expected to localize to membrane structures within these parasites. Although distinct membrane topologies are predicted, we present no evidence supporting the orientation of either antigen relative to membrane structures.

### PyE140 is expressed in *P*. *yoelii* sporozoites, liver, and blood stage parasites

Expression of the PyE140 antigen was confirmed in both *P*. *yoelii* sporozoites and blood stage schizonts by Western blot using antigen-specific mouse antisera (Fig 1B). In the *P*. *yoelii* sporozoite lysate, a predominant band was detected at approximately 82 kDa, while in *P*. *yoelii* schizonts, a smaller band was detected at approximately 53 kDa. A larger band at 82 kDa was also present in schizonts, as well as two additional smaller bands, though at less abundant levels, suggesting that PyE140 may be cleaved during blood stage parasite development. The PyE140 specificity of the polyclonal sera was confirmed, as no proteins were detected from uninfected mouse red blood cells.

IFA demonstrated that PyE140 is expressed in different stages of malaria parasites. PyE140 is distributed as discrete patches at both the apical and posterior ends of the sporozoite (Fig 1C). IFA also showed that PyE140 appears to accumulate on the parasitophorous vacuole membrane and developing merozoites of late (42 hour) liver stage parasites (Fig 1D) and developing merozoites in late blood stage schizonts (Fig 1E–1G). PyE140 expression was absent from 24 hour liver stage parasites, but was present at 48 hours (S3 Fig).

### PyE140 induces higher protection in mice against *P*. *yoelii* sporozoite challenge than PyCSP

**Fig 2:**
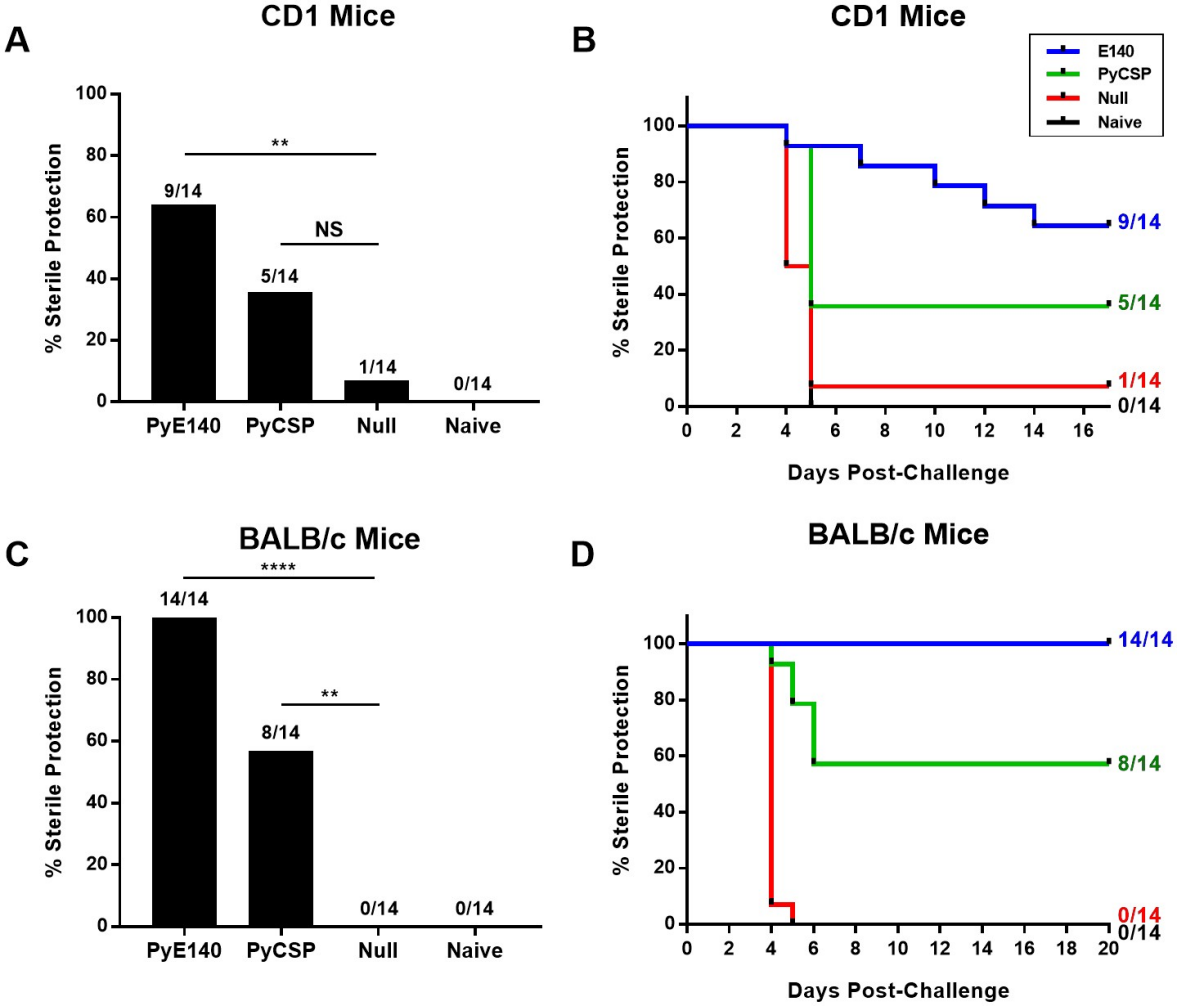
PyE140 vaccine is highly protective against *P*. *yoelii* sporozoite challenge in CD1 and BALB/c mice. Sterile protection and time to parasitemia were measured in mice after an intravenous challenge with *P*. *yoelii* 17XNL sporozoites. (A) Sterile protection. CD1 mice were immunized with DNA and HuAd5 vectors expressing PyE140na or PyCSP and challenged with 300 *P*. *yoelii* 17XNL sporozoites. A group of negative control mice immunized with DNA and HuAd5 null vectors that do not express a *P*. *yoelii* antigen and a group of naïve mice were also challenged. PyE140 sterilely protected 9/14 mice (64%), whereas PyCSP protected 5/14 mice (36%). It was notable that one PyE140-immunized mouse that became parasitemic self-cleared parasitemia by two days following the positive smear. This mouse was considered not protected and only the time to initial parasitemia is indicated in the graph. (B) Time to patency presented in a Kaplan-Meier plot. There was a delay to parasitemia in four of five PyE140-immunized CD1 mice that were not sterilely protected. There was no delay to parasitemia in the PyCSP-immunized mice that were not sterilely protected. Resolution of parasitemia is not indicated. Only the time of first observable parasitemia is indicated. Mice were smeared on days 4, 5, 6, 7, 10, 12, 14, and 17 after challenge. (C) Sterile protection. BALB/c mice were immunized with DNA and HuAd5 vectors expressing PyE140na or PyCSP and challenged with 100 Py17XNL sporozoites. A group of mice immunized with DNA and HuAd5 null vectors and a group of naïve mice were also challenged. PyE140 protected 14/14 mice (100%), whereas PyCSP protected 8/14 mice (57%). (D) Time to patency presented in a Kaplan-Meier plot. All of the PyE140-immunized BALB/c mice were sterilely protected. All PyCSP-immunized mice that were not sterilely protected became parasitemic by day 6 post-challenge. Mice were smeared on days 4, 5, 6, 7, 8, 10, 11, 13, 14, 15, and 20 after challenge. ****, **, and NS indicate *p*< 0.0001, *p*<0.01, and not significant by Fisher’s exact test, respectively.

The efficacy of PyE140 was compared with the known protective antigen, PyCSP, which historically has been the most protective malaria subunit vaccine in mouse studies [32, 33]. Outbred CD1 mice were immunized with DNA and HuAd5 vectors expressing native, non-codon-optimized PyE140 or PyCSP genes in a prime-boost regimen with a six week interval and challenged two weeks post-boost with an intravenous (IV) injection of 300 non-lethal *P*. *yoelii* 17XNL sporozoites. PyE140 sterilely protected 9/14 (64%) mice, whereas PyCSP protected 5/14 (36%) mice (Fig 2A). In addition to superior sterile protection, PyE140 immunization also resulted in delayed patency in 4/5 of the infected mice. Furthermore, one PyE140-immunized mouse was observed to clear parasitemia after a single positive blood smear. Conversely, PyCSP induced sterile protection with no delayed patency in non-protected mice and all non-protected mice remained parasitemic for two consecutive blood smears (Fig 2B). The DNA-HuAd5 PyE140na vaccine also sterilely protected 14/14 (100%) inbred BALB/c mice challenged with 100 *P*. *yoelii* 17XNL sporozoites, compared to 8/14 (57%) mice immunized with PyCSP (Fig 2C). No delay in patency was observed for either antigen as PyE140 sterilely protected all mice and all infected PyCSP-immunized mice became parasitemic on or before day 6 post-challenge (Fig 2D). The very high efficacy of PyE140 against malaria challenge was unexpected and warranted investigation of its protective mechanisms.

## PyE140-immunization elicits parasite-specific antibodies

**Fig 3:**
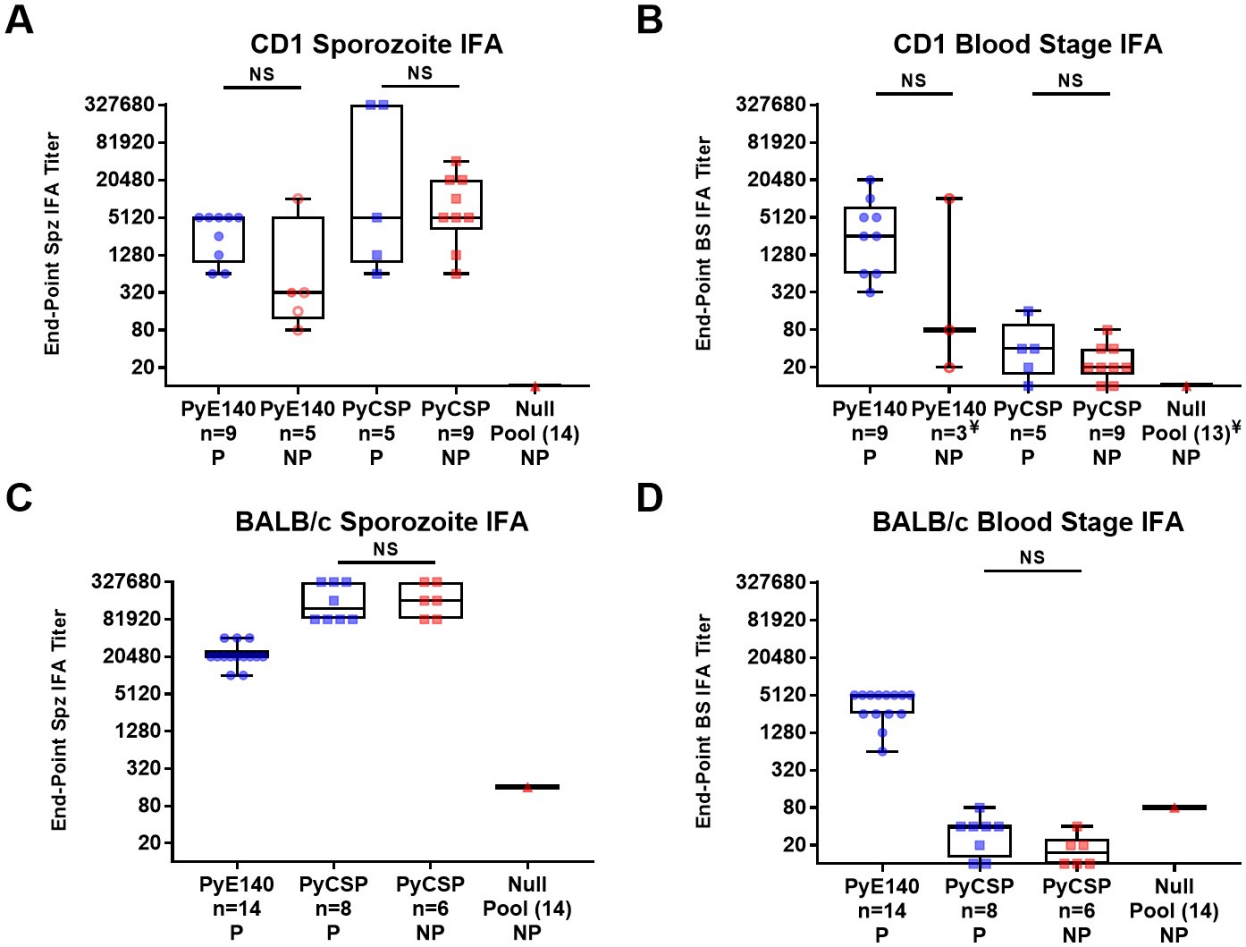
PyE140 immunization elicits parasite-specific antibodies. Antibody responses against (A) *P*. *yoelii* sporozoite and (B) blood stage schizonts were assessed with sera from CD1 mice immunized with DNA and HuAd5 vectors that express PyE140na or PyCSP, or null vectors that do not express a *P*. *yoelii* antigen. Antibody responses from the group of null mice were evaluated as a single pool. Antibody responses against (C) *P*. *yoelii* sporozoite and (D) blood stage parasites were also assessed with sera from BALB/c mice immunized with the same DNA and HuAd5 vectors. Sera from null-immunized mice were evaluated as a single pool. All sera were collected 1 week following the PyE140 HuAd5 boost, corresponding to 1 week before the challenge. The data are sorted based on protection status. NS indicates not significant by Mann-Whitney. P, Protected. NP, Not Protected. ¥, Sera from all mice were not available at the time of testing. Spz, Sporozoite. BS, Blood stage. Blue symbols indicate protected mice and red symbols indicate non-protected mice. A solid red circle indicates a PyE140-immunized mouse that became parasitemic by day 6 after challenge and open red circles indicate PyE140-immunized mice that became parasitemic on day 7 or later after challenge.

PyE140-specific antibodies induced in the protection studies shown in Fig 2 recognized both *P*. *yoelii* sporozoites and *P*. *yoelii* blood stages (Fig 3). With the exception of one mouse, sera from PyE140-protected CD1 mice had higher IFA titers to *P*. *yoelii* sporozoites (Fig 3A) and *P*. *yoelii* blood stages (Fig 3B) than sera from non-protected mice. However, when all mice are included in the analysis, the difference between the titers of the protected and non-protected mice was not statistically significant. Interestingly, the non-protected mouse with the highest PyE140 antibody titer is the same mouse described above that cleared parasitemia after a single positive blood smear on day 12 post-challenge. As expected, sera from PyCSP-immunized CD1 mice had high titers to *P*. *yoelii* sporozoites and low titers to *P*. *yoelii* blood stages. The *P*. *yoelii* sporozoite IFA titers of sera from the protected and non-protected PyCSP-immunized CD1 mice were comparable. Similar results were also observed in the BALB/c mice, although all PyE140-immunized BALB/c mice were protected (Fig 3C and 3D).

### Passive transfer of PyE140 immune serum partially protects recipient mice

**Fig 4:**
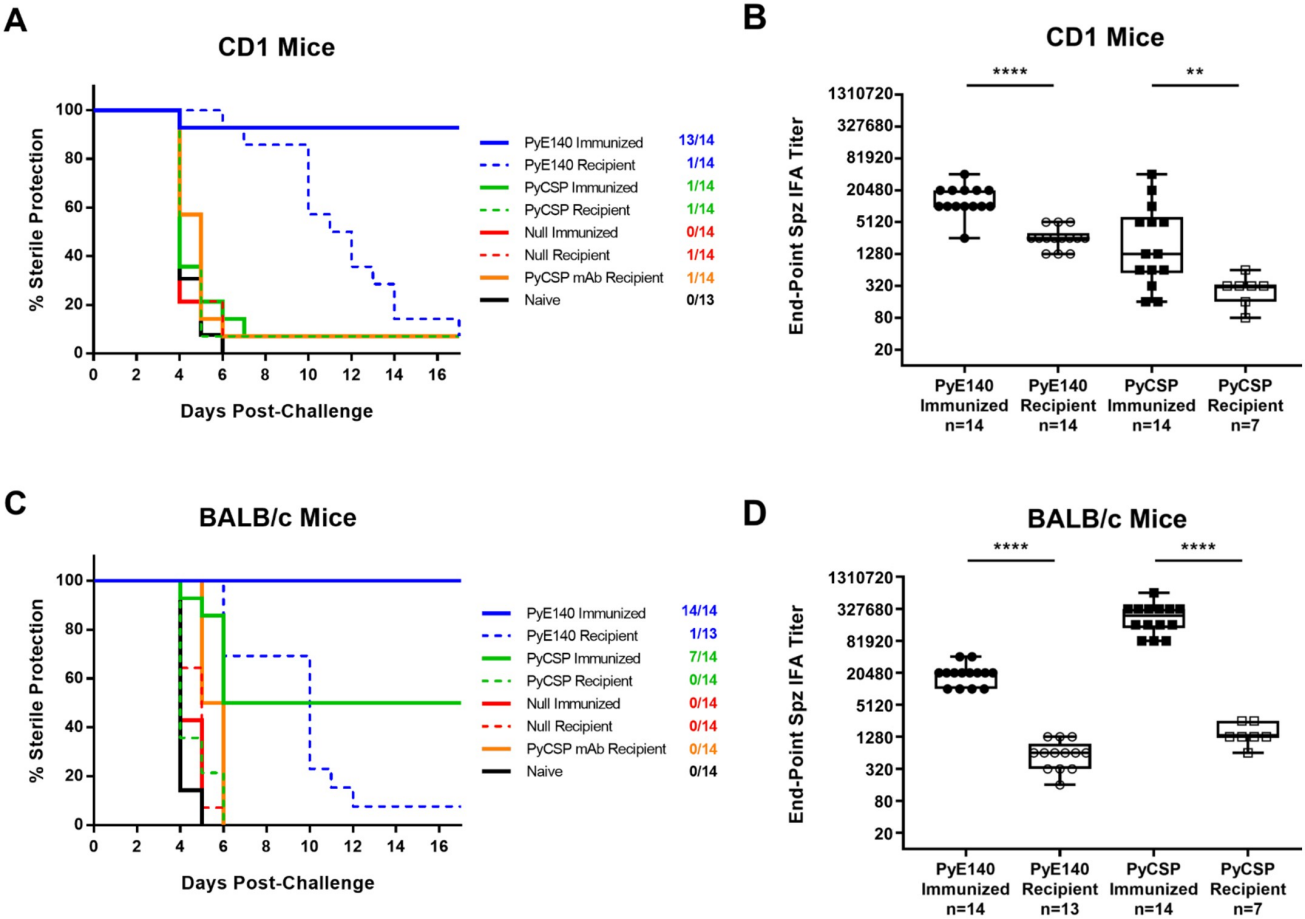
PyE140-specific antibodies delay patency. Time to parasitemia following an intravenous challenge with *P*. *yoelii* sporozoites and antibody titers were assessed in mice passively transferred with sera from mice immunized with DNA and HuAd5 vectors expressing PyE140na or PyCSP. (A) There was a delay to parasitemia in CD1 mice passively transferred with sera from mice immunized with PyE140na (*p*<0.01, Mantel-Cox), but not PyCSP, when compared to recipients of sera from null-immunized mice. Mice were smeared on days 4, 5, 6, 7, 10, 11, 12, 13, 14, and 17 post-challenge. (B) Antibody responses against *P*. *yoelii* sporozoites were assessed in immunized mice one week prior to challenge and in passively transferred CD1 mice approximately five to six hours before challenge. Antibody titers were significantly lower in the passively transferred mice than the immunized mice. (C) There was a delay to parasitemia in BALB/c mice passively transferred with sera from mice immunized with PyE140na (*p*<0.0001, Mantel-Cox), but not PyCSP, when compared to recipients of sera from null-immunized mice. Mice were smeared on days 4, 5, 6, 7, 10, 11, 12, 13, 14, and 17 post-challenge. (D) Antibody responses against *P*. *yoelii* sporozoites were assessed in immunized mice 1 week prior to challenge and passively transferred BALB/c mice on the day of challenge. Titers were significantly lower in the passively transferred mice than the immunized mice. **** indicates a statistical significance of *p*<0.0001 and ** indicates a statistical significance of *p*<0.01 by Mann-Whitney.

A serum transfer experiment was performed to determine if PyE140 antibodies were sufficient to protect CD1 mice against a *P*. *yoelii* sporozoite challenge. After passive transfer of serum from PyE140-immunized CD1 mice, all recipient mice remained uninfected for six days post-challenge followed by a gradual decline in protection until day 17, when 1/14 (7%) mice remained uninfected (Fig 4A). In contrast, after passive transfer of either serum from PyCSP-immunized mice or NYS1, a monoclonal antibody (mAb) to PyCSP, 13/14 (93%) mice became infected by day 6 post-challenge. Negative control mice all became infected 4–6 days after challenge. As positive controls, the DNA-HuAd5 PyE140na vaccine again protected a higher percentage of mice than the PyCSP vaccine (93% vs. 7%).

Anti-sporozoite IFA titers after passive transfer and before challenge were significantly lower in the recipient mice than the mice that were directly immunized with PyE140 (Fig 4B), suggesting that sterilizing levels of antibodies were not achieved in the recipient mice, resulting in a gradual breakthrough of parasitemia for 17 days post-challenge. Although antibody titers in the directly immunized PyCSP mice were also higher than in the PyCSP recipient mice (Fig 4B), no delay was observed after transfer of PyCSP antisera or NYS1.

In BALB/c mice, a similar outcome was observed; passive transfer of serum from PyE140-immunized mice significantly delayed the onset of parasitemia until day 12 when 1/14 (7%) mice remained uninfected (Fig 4C), whereas mice passively transferred with serum from PyCSP-immunized mice or NYS1 were not protected and there was no delay to parasitemia (Fig 4C). As was observed in CD1 mice, levels of PyE140 antibodies after passive transfer were significantly lower than after direct PyE140 immunization (Fig 4D). We interpret these results to suggest that antibodies to PyE140 more effectively mediate protection against *P*. *yoelii* malaria than PyCSP-specific antibodies.

### PyE140 immunization generates high frequency PyE140-specific CD4^+^ and CD8^+^ T cells that are not required for protection

**Fig 5:**
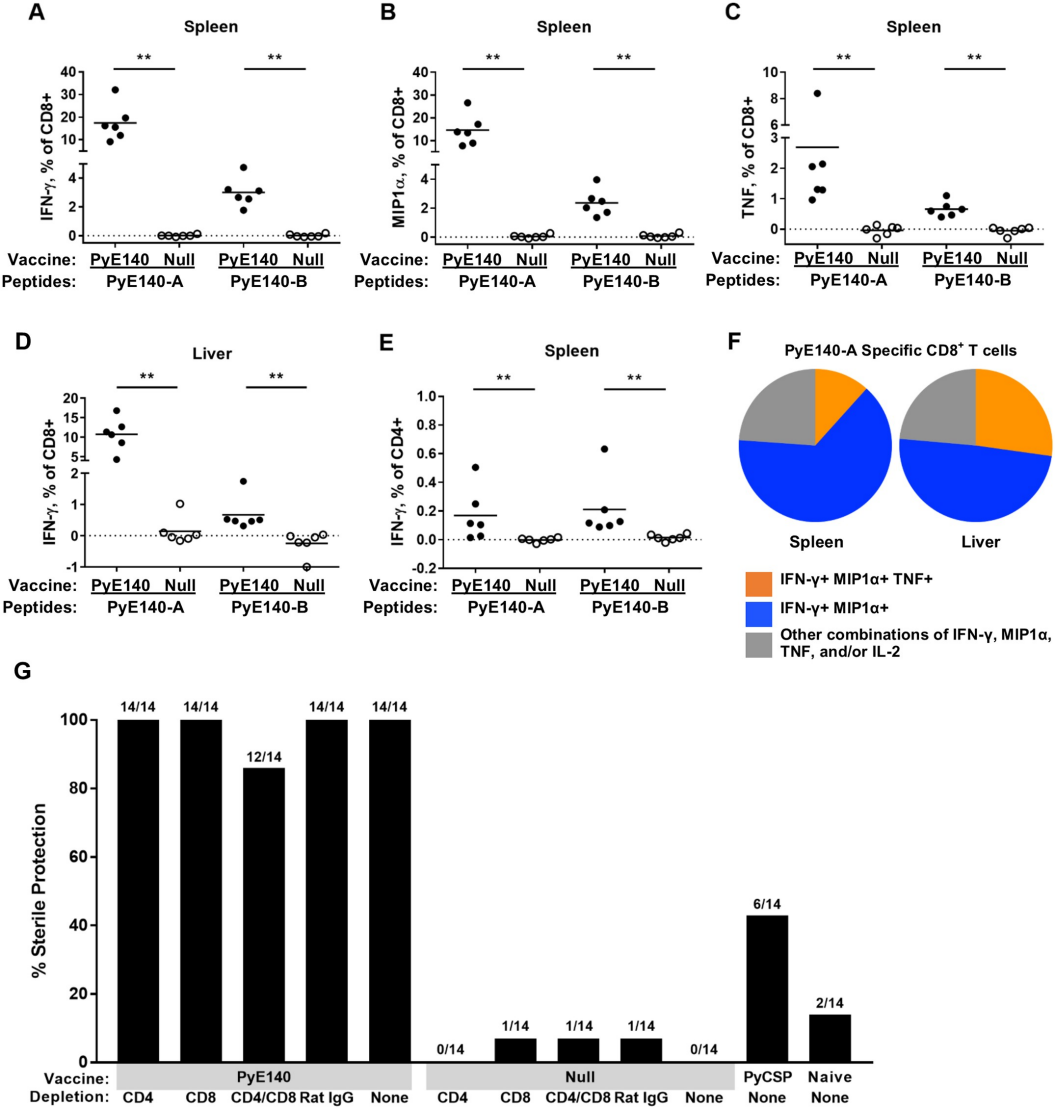
PyE140 immunization induces high frequency multifunctional CD8^+^ T cells in the spleen and liver of immunized mice which are not required for protection. CD1 mice were immunized with DNA and HuAd5 vectors expressing PyE140na or null vectors. Two weeks after the boost, lymphocytes isolated from spleen and liver were stimulated with either peptide pool PyE140-A or PyE140-B for 4 hours for intracellular cytokine staining and subsequent analysis by flow cytometry. Responses are background subtracted using the DMSO negative control stimulations. The frequency of CD8^+^ T cells from spleen producing (A) IFN-γ, (B) MIP1α, and (C) TNF are shown. (D) The frequency of CD8^+^ T cells from liver producing IFN-γ are shown. (E) The frequency of CD4^+^ T cells from spleen producing IFN-γ are shown. (F) Pie charts show the proportion of splenic PyE140-A-specific CD8^+^ T cells producing either two (IFN-γ and MIP1α—blue) or three (IFN-γ, MIP1α, and TNF—orange) cytokines. Gray indicates the remaining proportion of the response, which is predominately composed of single producers of either IFN-γ or MIP1α. The gating strategies for identification of antigen-specific cells are shown in S4 Fig. (G) CD1 mice immunized with either PyE140na or null vectors as above were depleted of CD4^+^ and/or CD8^+^ T cells and challenged with *P*. *yoelii* sporozoites. Sterile protection was assessed by blood smears. Mice were smeared on days 4, 5, 6, 7, 8, 10, 11, 13, 14, 15, and 19 post-challenge. Depletion efficiency is shown in S6 Fig. ** indicates *p*<0.01 by Mann-Whitney.

Groups of six CD1 mice were immunized with DNA and HuAd5 vectors expressing PyE140na or null vector controls to evaluate the cellular responses induced by PyE140 immunization. Spleens and livers were harvested two weeks after the HuAd5 boost. Heterologous DNA-HuAd5 PyE140na prime-boost immunization induced high frequencies of PyE140-specific CD8^+^ T cells in both the spleen and liver that produced interferon-gamma (IFN-γ), macrophage inflammatory protein 1 alpha (MIP1α), and tumor necrosis factor (TNF) in response to stimulation with peptide pools representing the N-terminal half (PyE140-A) or the C-terminal half (PyE140-B) of the PyE140 protein (Fig 5A–5D; S5E and S5F Fig). Frequencies of CD8^+^ T cells producing IFN-γ when stimulated with the PyE140-A peptide pool ranged from 9.2–32% of CD8^+^ T cells in the spleen (Fig 5A) and from 4.3–17% of CD8^+^ T cells in the liver (Fig 5D). CD4^+^ T cells producing IFN-γ or TNF were observed at low frequency in the spleen, but were not distinguishable above background in the liver (Fig 5E and Panels 5C, 5H, and 5J in S5 Fig). Only one of six mice had splenic CD4^+^ T cell MIP1α responses to PyE140-A exceeding three standard deviations above the mean of the null-immunized mice (Panel B in S5 Fig). IL-2 was also measured, but was not detected above background in either CD4^+^ or CD8^+^ T cells (Panels A, D, G, and K in S5 Fig). The majority of CD8^+^ T cells responding to either PyE140 pool A or B were multifunctional, producing both IFN-γ and MIP1α, with or without TNF (Fig 5F).

To determine whether these CD4^+^ and/or CD8^+^ T cells directly mediate PyE140-induced protection, we performed *in vivo* depletion of these cell populations in mice immunized with DNA and HuAd5 vectors expressing PyE140na or null vectors (Fig 5G and S6 Fig). In this study, the PyE140 vaccine protected 100% of the non-depleted mice, 100% of the CD4^+^ T cell depleted mice, 100% of the CD8^+^ T cell depleted mice, and 84% of the CD4^+^ and CD8^+^ T cell depleted mice, suggesting that despite the high frequency and multifunctionality of the PyE140-induced CD8^+^ T cell responses, these cells are not required for protection. Although evaluation of the CD4^+^ and CD8^+^ T cell frequencies in the spleens of some of the depleted mice indicated that the CD4^+^ T cells were not completely depleted (S6 Fig), we observed no significant reduction in the proportion of protected mice. Due to the low frequency of PyE140-induced CD4^+^ T cells and substantial depletion among some mice, we conclude that CD4^+^ T cells likely do not directly mediate protection. Nonetheless, the CD4^+^ T cell depletion results should be regarded with care due to the difficulty in depleting this population from tissues.

### PyE140 immunization confers long-term sterile protection and induces durable antibody responses which are associated with protection

**Fig 6:**
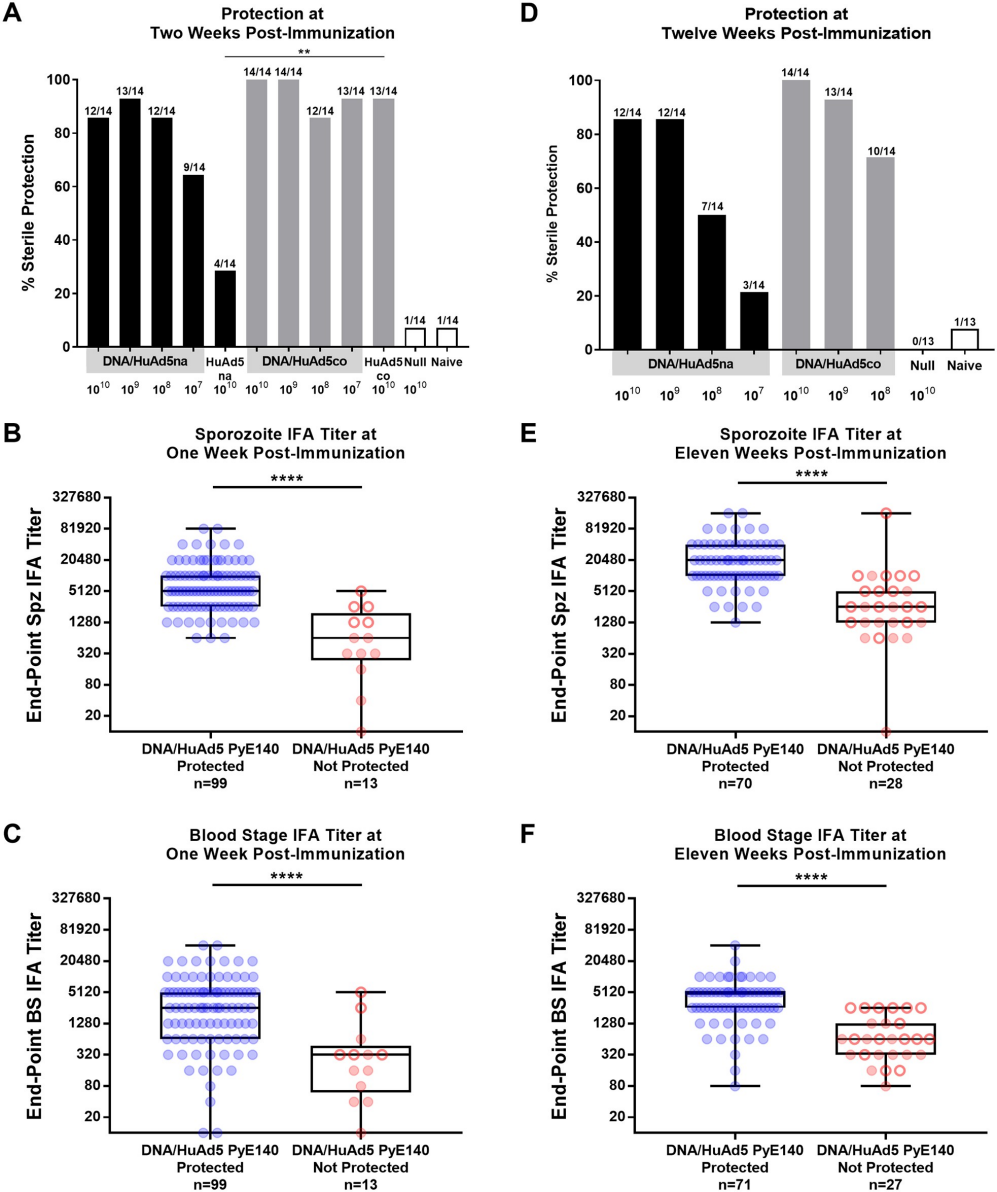
PyE140 immunization confers long-term sterile protection and induces durable antibody responses which are associated with protection. CD1 mice were immunized with a single dose of HuAd5-PyE140na or HuAd5-PyE140co, or with a prime-boost regimen consisting of a DNA-PyE140na prime and a HuAd5-PyE140na or HuAd5-PyE140co boost. The mice were challenged with 300 *P*. *yoelii* sporozoites at 2 weeks or 12 weeks post-boost. A group of mice immunized with DNA and HuAd5 null vectors and a group of naïve mice were also challenged in each study. (A) Sterile protection of mice challenged 2 weeks post-boost. Mice were smeared on days 4, 5, 6, 7, 8, 11, 13, and 15 post-challenge. (B-C) Anti-Py sporozoites and blood stage IFA titers of mice 1 week post-boost (1 week before challenge), grouped by challenge outcome. (D) Sterile protection of a second set of mice challenged 12 weeks post-boost. Mice were smeared on days 4, 5, 6, 7, 10, 12, 14, 17, and 19 post-challenge. (E-F) Anti-Py sporozoites and blood stage IFA titers of mice 11 weeks post-boost (1 week before challenge), grouped by challenge outcome. Blue symbols indicate mice that were sterilely protected. Red solid symbols indicate mice that first became parasitemic from days 4 to 6 post-challenge. Red open symbols indicate mice with delayed parasitemia that first became blood smear positive at day 7 or later post-challenge. **** indicates *p*<0.0001 by Mann-Whitney.

To optimize the efficacy of the PyE140 vaccine, we generated a new HuAd5-PyE140 vector encoding a full length synthetic PyE140 gene, codon-optimized for mammalian expression (PyE140co), rather than the native PyE140 gene (PyE140na). The HuAd5-PyE140co vector expresses significantly more PyE140 *in vitro* than HuAd5-PyE140na (S7 Fig). The efficacy of the new codon-optimized HuAd5-PyE140co vector was evaluated in CD1 mice in single dose and prime-boost regimens. Efficacy of the DNA-PyE140na-HuAd5-PyE140co prime-boost vaccine was consistently the same or greater than the DNA-PyE140na-HuAd5-PyE140na prime-boost vaccine at multiple HuAd5 doses (Fig 6A), and 100% efficacy was achieved with the HuAd5-PyE140co vector at 10^9^ and 10^10^ particle units (pu) doses (Fig 6A). Remarkably, 93% sterile protection was observed with only a single dose (1 x 10^10^ pu) of the HuAd5-PyE140co vector compared to 29% with a single dose of the HuAd5-PyE140na vector. This was a highly unexpected result, as a prior study with groups of BALB/c mice each immunized with a single dose (1 x 10^10^ pu) of six different HuAd5 vectors expressing different non-CSP codon-optimized *P*. *yoelii* vaccine genes resulted in no protection, as measured by reduction in liver stage burden [34]. In our experience, previous studies with non-CSP antigens have not yielded such high level sterile protection, even with prime-boost regimens [19, 35]. Similar to the first PyE140 protection study, we again observed that some mice (three) cleared parasitemia within 2–4 days and remained blood smear negative for the remainder of the study.

Evaluation of antibody titers by sporozoite and blood stage IFA showed that parasite-specific antibodies against both sporozoites and blood stage schizonts were increased in protected mice compared to non-protected mice (Fig 6B and 6C). The groups immunized with HuAd5-PyE140co had either comparable or higher IFA titers to *P*. *yoelii* sporozoites and blood stages than the groups immunized with HuAd5-PyE140na, most dramatically in the low dose 10^7^ pu prime-boost group (Panels A and C in S8 Fig). Since IFAs were performed with sera taken one week after the HuAd5-PyE140 immunization, the IFA titers for the single dose HuAd5-PyE140 groups may not accurately represent the titers at the time of challenge (two weeks post-immunization), as boosted responses increase sooner than those following initial exposure to antigen.

PyE140 vaccination also induced long-term sterile protection. CD1 mice primed with DNA-PyE140na and boosted with various doses of HuAd5-PyE140na or HuAd5-PyE140co were sterilely protected when challenged 12 weeks post-boost (Fig 6D). In all comparable groups, the DNA-HuAd5-PyE140co vaccine protected a greater percentage of mice than the DNA-HuAd5-PyE140na vaccine when challenged 12 weeks post-boost, though due to the high efficacy of the native vaccine, this difference was not statistically significant (Fig 6D). Again, we observed three mice that cleared parasitemia after a single positive blood smear. We also observed high and sustained IFA antibody responses to *P*. *yoelii* sporozoites and blood stages at eleven weeks post-boost (Fig 6E and 6F), consistent with the high level of protection at twelve weeks and the prior evidence indicating a role for antibody mediated protection by PyE140 immunization. IFA titers against both sporozoites and blood stage schizonts at eleven weeks post-boost were again increased among protected mice compared to non-protected mice (Fig 6E and 6F).

### PyE140 protects against blood stage parasite challenge

**Fig 7:**
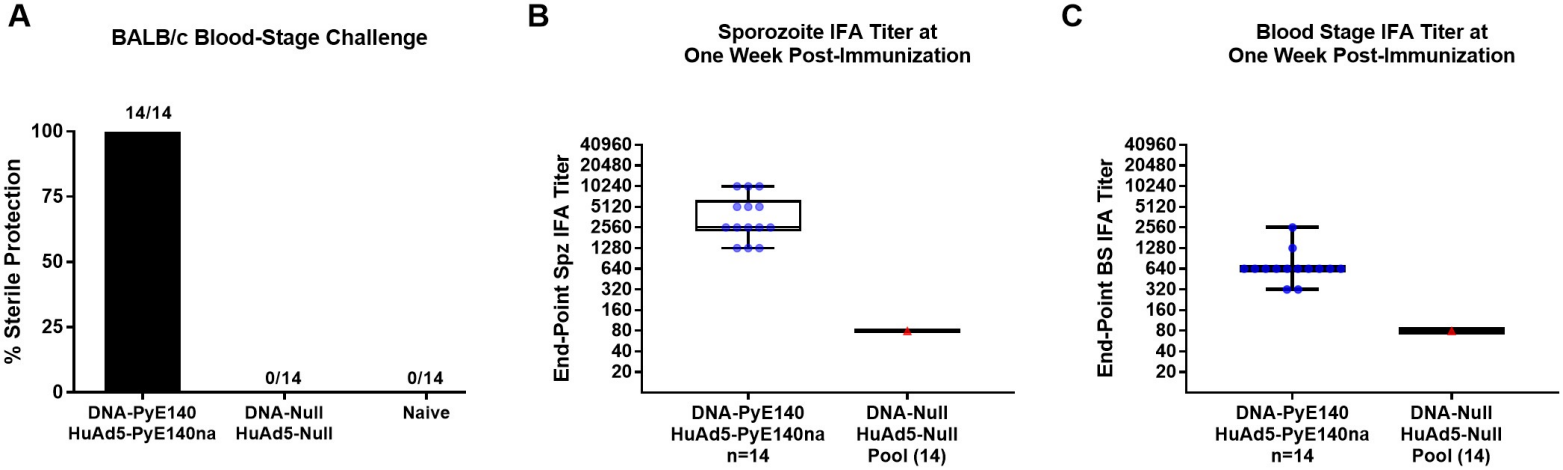
PyE140 immunization protects against blood stage challenge. BALB/c mice were immunized with DNA and HuAd5 vectors encoding PyE140na. Additional mice immunized with DNA and HuAd5 null vectors and naïve mice were included as negative controls. (A) Mice were challenged intravenously in the lateral tail vein with 10,000 *P*. *yoelii* 17XNL-infected red blood cells two weeks after the final immunization. Mice were smeared on days 2, 3, 4, 5, 7, 9, 11, and 14 post-challenge. Negative control (null-immunized and naïve) mice all became positive by day 3 post-challenge. Antibody titers from sera drawn one week post-boost were determined by IFA against (B) sporozoites and (C) blood stage parasites.

Because of the measurable delay in the onset of parasitemia after sporozoite challenge in previous experiments (Figs 2B, 4A and 4C), a study was undertaken to assess the ability of PyE140 to protect mice against a blood stage challenge. All BALB/c mice immunized with DNA and HuAd5 vectors expressing PyE140na were sterilely protected (Fig 7), demonstrating that PyE140 blood stage parasites are a target of PyE140 vaccine induced protection.

**Fig 8:**
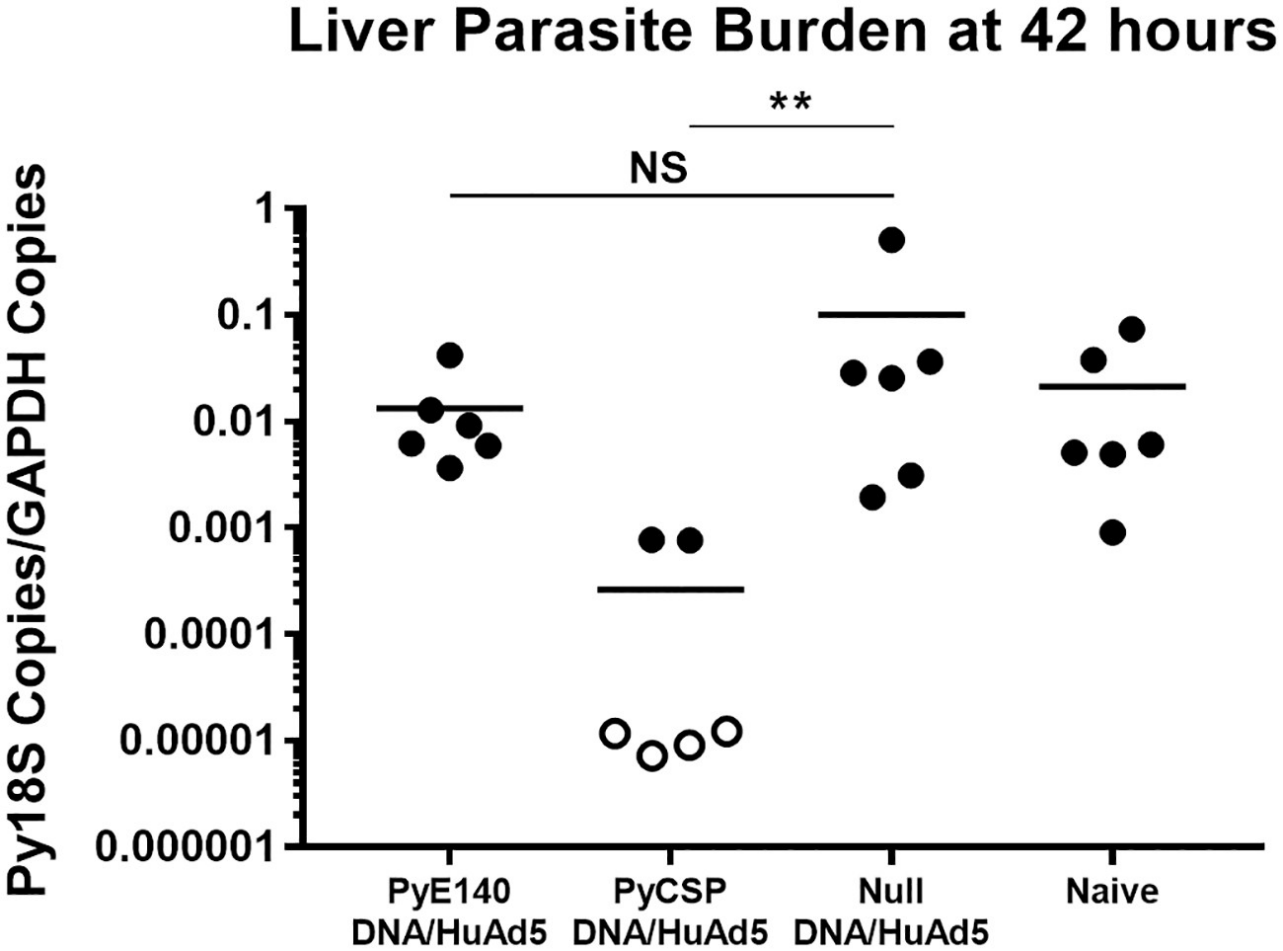
PyE140 immunization does not reduce liver stage parasite burden. Eleven week old CD1 mice were immunized with DNA and HuAd5 vectors expressing either PyE140 or PyCSP at weeks 0 and 6, respectively. The HuAd5-PyE140 vector encoded the codon-optimized PyE140co gene. Groups of null immunized and naïve mice were also included. Two weeks after the boost, mice were challenged with 20,000 *P*. *yoelii* 17XNL sporozoites by subcutaneous injection. Livers were harvested 42 hours after challenge for evaluation of Py18S RNA copies/murine GAPDH RNA copies by RT-qPCR. Filled symbols represent detectable Py18S RNA copies, and open symbols represent Py18S RNA copies below the limit of detection (10 copies of Py18S RNA) and are plotted at ½ the limit of detection (5 copies of Py18S RNA). ** indicates *p*<0.01 and NS indicates not significant by Mann-Whitney.

Because mice were completely protected against a blood stage challenge and PyE140 is expressed in sporozoites, liver and blood stage parasites, we sought to determine whether PyE140 immunization also acted against pre-erythrocytic stage parasites or if protection could be due to blood stage inhibition alone. CD1 mice (n = 6) immunized with DNA-HuAd5 prime-boost vaccines expressing either PyE140co or PyCSP were evaluated for liver stage parasite burden by RT-qPCR 42 hours after challenge (Fig 8). Because antibodies may act against sporozoites in the skin as parasites translocate from the skin to the bloodstream, we used subcutaneous challenge with 20,000 *P*. *yoelii* 17XNL sporozoites. While PyCSP immunization resulted in a significant decrease in liver stage burden with four mice having undetectable Py18S RNA, there was no significant reduction in liver stage burden in mice immunized by PyE140. As we were concerned that this high dose of sporozoites could overwhelm the protective immune response, we also performed a parallel subcutaneous challenge with 5,000 sporozoites, and obtained a similar result (S9 Fig). IFA was performed on pooled sera for the PyE140co immunized mice (n = 12) and the end-point dilution was 20,480 for recognition of sporozoites and 2,160 for recognition of blood stage schizonts. These titers are consistent with those observed in prior experiments, confirming that antibodies to both stages were induced by the vaccine, but only those to PyE140 expressed in blood stages were protective. Overall, these data demonstrate that the high level of protection elicited by PyE140 immunization is mediated against parasites maturing after the late (42 hour) liver stage, which could include egress of merozoites from the liver, red blood cell invasion, or egress of merozoites from red blood cells.

### Increased PyE140 vaccine-induced antibody titers are associated with both sterile protection and delay of parasitemia in non-protected mice

**Fig 9:**
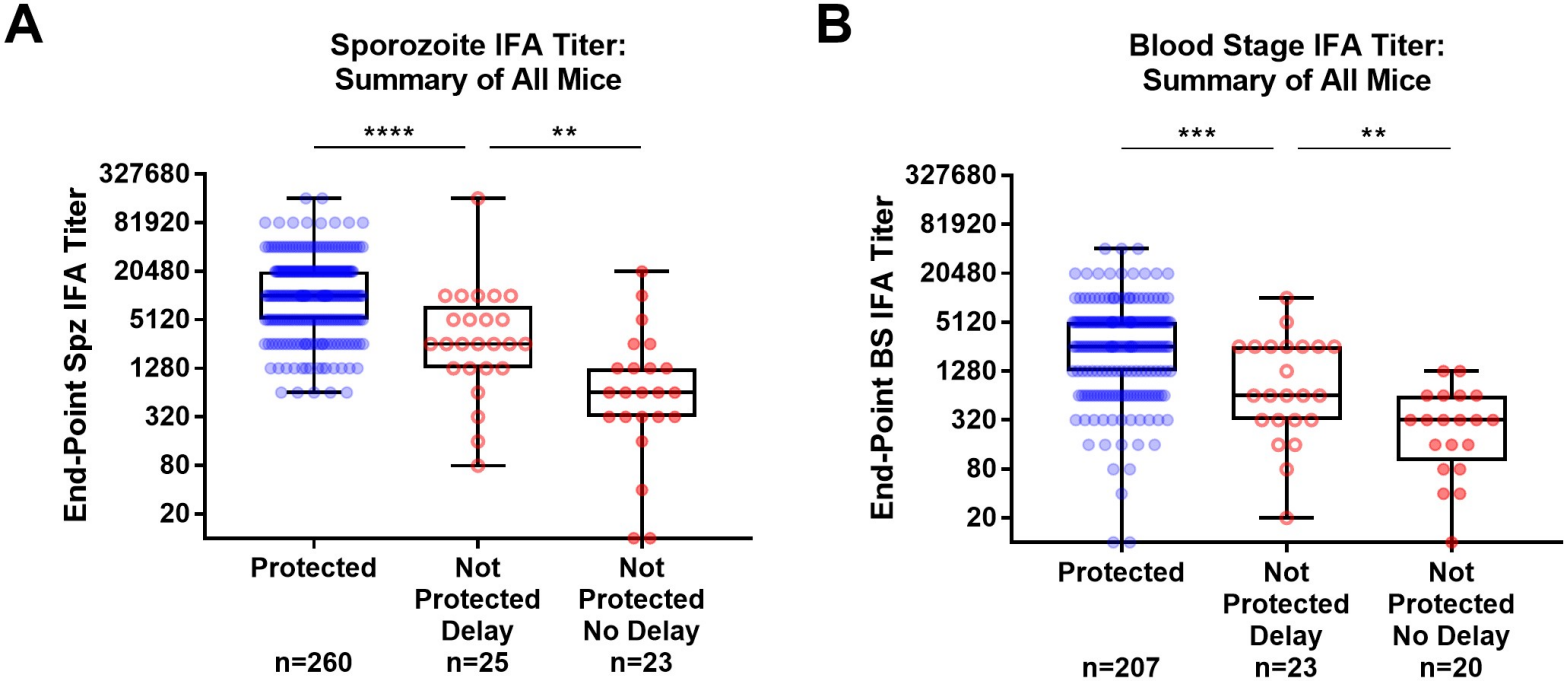
Increased PyE140 vaccine-induced antibody titers are associated with both sterile protection and delay of parasitemia in non-protected mice. All sporozoite and blood stage IFA end-point titers for mice immunized against PyE140 by DNA-HuAd5 prime-boost and evaluated by sterile protection, for which individual IFAs were performed, are shown. These include the sporozoite and blood stage IFA titers shown above in Figs 3, 6 and 7. Sporozoite IFAs from Fig 4 were also included for the immunized mice only; blood stage IFAs were not performed and are therefore not included. Sera recipients from this study were not included. Sporozoite IFAs on individual mice were also performed for animals in Group 1 (CD4 depleted) and Group 3 (CD4/CD8 depleted) from Fig 5G, and are therefore included. IFAs for individual mice were not performed on the remaining groups in this study and were therefore not included. (A) Anti-Py sporozoite and (B) anti-Py blood stage IFA titers of mice 1 week before challenge are grouped by challenge outcome. Blue symbols indicate mice that were sterilely protected. Red solid symbols indicate mice that first became parasitemic from days 4 to 6 post-challenge. Red open symbols indicate mice with delayed parasitemia that first became blood smear positive at day 7 or later post-challenge. ****, ***, and ** indicate *p*<0.0001, *p*<0.001, and *p*<0.01 by Mann-Whitney.

To further evaluate the association of antibody titers with delayed parasitemia, we performed an analysis of antibody data from all PyE140 immunized and challenged mice evaluated by blood smear in all of the studies described above (Fig 9). In general, mice that had a delay in parasitemia (detected at day 7 or later post-challenge) had increased antibody titers to both sporozoite and blood stage schizonts compared to mice that became parasitemic by day 6 post-challenge. In addition, mice that were sterilely protected following challenge had increased antibody titers compared to mice that had delayed parasitemia. These data suggest that PyE140 immunization induced antibody-mediated protection against blood stage parasites.

### Limited *P*. *falciparum* E140 sequence variation supports antibody cross-reactivity

**Fig 10:**
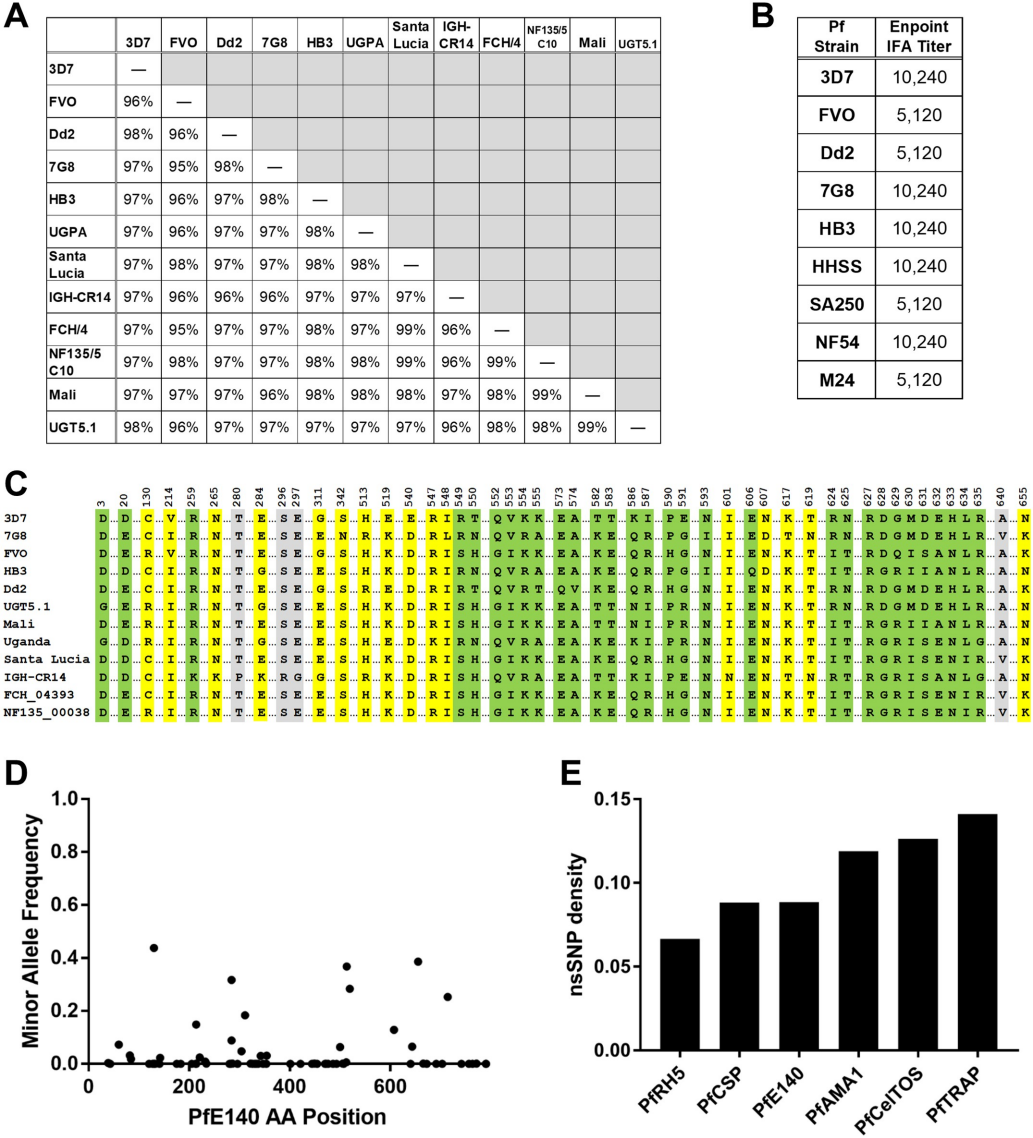
PfE140 sequence diversity and antibody cross-recognition. (A) Amino acid homology of PfE140 protein from 12 lab-adapted *P*. *falciparum* strains. PfE140 amino acid sequences as listed in the Plasmodium 100 Genomes initiative, Broad Institute, (broadinstitute.org) were aligned by BLAST. The full length Dd2 sequence was obtained from PlasmoDB. (B) Immunofluorescence cross-reactivity of PfE140 (3D7) serum to schizonts of eight laboratory-adapted strains. Late schizonts were cultured for 42 hours post-invasion, Percoll enriched, and probed with mouse polyclonal serum induced with a single dose of HuAd5-PfE140. IFA titers are expressed as end-point reactivity in a serial serum dilution. All parasites that reacted with serum showed a similar PfE140 fluorescence pattern (S10 Fig). (C) The incidence and position of all PfE140 sequence variations for the same twelve *P*. *falciparum* laboratory-adapted strains shown in Fig 10A. Sixteen of the 50 variant positions were identified as circulating nsSNPs (yellow highlighting) among over 3,000 *P*. *falciparum* samples collected from 23 countries (MalariaGEN *P*. *falciparum* Community Project, www.malariagen.net/projects/parasite/pf, accessed on 12/7/2015 and 10/8/2019). Variation at positions that were invariant in the MalariaGEN *P*. *falciparum* Community Project are assumed to be due to culture adaptation (green highlighting). Four positions had nsSNPs identified in the MalariaGEN *P*. *falciparum* Community Project, but the substitution was for a different amino acid than the substitution identified among the laboratory isolates (gray highlighting). (D) The minor allele frequency and positions of all nsSNPs for the PfE140 gene (MalariaGEN Community Project, www.malariagen.net/projects/parasite/pf, data accessed 10/8/2019). (E) The nsSNP density of six malaria vaccine genes. The nsSNP density was determined by dividing the number of variant positions by the number of total amino acids in the protein.

Antigen polymorphism is a critical feature for any malaria vaccine. Importantly, there is a high degree of amino acid conservation in the PfE140 protein from 12 geographically diverse, laboratory-adapted *P*. *falciparum* strains (Fig 10A). This homology was consistent with the strong cross-reactivity of sera raised against a PfE140 (clone 3D7) vaccine with mature schizont stages of eight different laboratory-adapted *P*. *falciparum* strains (Fig 10B). These results indicate that the low variation in PfE140 proteins does not prevent the PfE140 (3D7)-raised antibody from recognizing diverse strains. Sequence alignment of the PfE140 protein from the 12 strains compared in Fig 10A, including five tested for antibody cross-reactivity in Fig 10B, shows that variations occurred at 50 different positions (of 791 total, Fig 10C). Most of the amino acid variations among the laboratory isolates were not identified among non-synonymous single nucleotide polymorphisms (nsSNPs) among circulating parasite sequences (Fig 10D), suggesting that much of the variation among the laboratory isolates is due to culture adaptation (Fig 10C). Furthermore, an in depth analysis of nsSNPs for PfE140 among 3,441 circulating parasites revealed a low frequency of SNPs for the entire protein, with only 13 positions having variants present at above 5% (Fig 10D). A total of 75 nsSNPs at 69 positions were identified for the 791 codon gene, which is a lower nsSNP density than several other malaria vaccine candidates, such as PfAMA1, PfCelTOS, and PfSSP2/TRAP, with only PfCSP and PfRH5 being either comparable or more conserved than PfE140 (Fig 10E). The high level of conservation, low frequency of nsSNPs, and strain-transcending antibody cross-reactivity strongly suggest that PfE140 could elicit broadly reactive immunity among circulating strains, which is critical for a successful malaria vaccine.

## Discussion

Efforts to discover, characterize, and evaluate new malaria vaccine antigens have frequently utilized proteomic and transcriptomic abundance, antigenic characteristics, and immunogenicity in endemic areas or animal models as selection criteria. We have evaluated potential vaccine candidates by using vector-based vaccines expressing the corresponding *P*. *yoelii* orthologs to protect mice against a stringent *P*. *yoelii* sporozoite challenge [19]. The assumption of this approach is that if antigens can induce sterilizing protection in a rodent model, it would strongly support evaluation of orthologous antigens for development of a single or multivalent subunit vaccine against *P*. *falciparum* malaria. Among the vaccine antigens that we have evaluated thus far, none have shown the unique protective profile seen with E140.

PyE140-based vaccines in the *P*. *yoelii* mouse model induce consistent, high level protection. Overall, PyE140-based vaccines, including suboptimal vaccine doses, sterilely protected 319 of 378 (84%) inbred and outbred mice against *P*. *yoelii* sporozoite and blood stage challenges. Immunization by a DNA-HuAd5 (10^10^ pu) prime-boost regimen resulted in protection of 184 of 196 (94%) mice. Initial studies were performed with a heterologous prime-boost immunization regimen with DNA and HuAd5 vectors expressing the native PyE140 gene, PyE140na. Subsequent studies utilized a HuAd5 vector that expressed a mammalian codon-optimized PyE140 gene, PyE140co, which increased *in vitro* antigen expression. Protection was significantly increased when HuAd5-PyE140co was administered as a single dose compared to a single dose of HuAd5-PyE140na. Protection was also increased when the HuAd5-PyE140co vaccine was used in a DNA-HuAd5 prime-boost regimen compared to the same regimen using the HuAd5-PyE140na vaccine, yet the increase was not significant, as even the HuAd5-PyE140na vaccine expressing low amounts of protein consistently induced high level protection in the prime-boost regimen.

Overall, there is a strong correlation between antibody titers against either sporozoite or schizonts and protection among PyE140-immunized mice. Consistent with this association, we also noted that the delay to patency was associated with low antibody titers, suggesting that high antibody titers are required for blocking or delaying parasite expansion in the blood. Many of the PyE140-immunized mice that were not sterilely protected in these studies exhibited a significant delay in the onset of parasitemia. In fact, 30 of the 59 mice that were not sterilely protected, including those that received a single dose of HuAd5-PyE140, had a delay in patency relative to naïve and null-immunized mice. Furthermore, seven of these mice controlled and eventually cured the infection after exhibiting very low parasitemia for 2–3 days. This unusual occurrence led us to test the fitness of the parasites circulating in the mice with these very low, transient parasitemias. We transferred diluted blood containing approximately 10,000 infected red blood cells from a PyE140-immunized mouse that maintained very low parasitemia (0.1–0.5% infected RBCs) into a naïve mouse. The recipient mouse became heavily infected within 4 days, indicating that the parasites in the transiently infected donor mice were fit, but the infection could not fully develop, likely due to the suppressive activity of the PyE140 antibodies.

Although most of the PyE140 protection studies were performed in sporozoite challenged mice, we confirmed that the parasite killing observed in our studies does not take place at the pre-erythrocytic (sporozoite and liver) stages, but after the liver stage as measured by RT-qPCR on livers removed 42 hours after a subcutaneous sporozoite challenge. Furthermore, although PyE140 immunization led to extremely high cellular immune responses in the spleen and liver, T cell depletion of mice prior to challenge indicated that CD4^+^ and CD8^+^ T cells do not mediate protection. Additional evidence indicating that PyE140 antibodies targeting blood stage parasites are responsible for protection is that 100% of PyE140-immunized mice were protected against a blood stage challenge.

It is important to note that there is still some possibility that following either intradermal or mosquito bite challenge, antibodies could have an additional effect on sporozoites before entering the blood vessels due to increased migration and duration in the skin. While sterile protection observed in our studies following intravenous challenge appears to be entirely due to antibody inhibition of blood stages, this may not preclude additional inhibitory antibody activity against sporozoites in the skin.

Another notable feature of PyE140 protection is its longevity. Protection was maintained at 86% or greater at 12 weeks post-immunization among four groups of mice immunized by DNA-HuAd5 prime-boost regimens with a HuAd5-PyE140na or HuAd5-PyE140co boost of 10^9^ or 10^10^ pu. Antibody responses were either similar or higher before the twelve week challenge compared the two week challenge, except for the highest dose of the HuAd5-PyE140co vaccine boost, where the titer prior to the twelve week challenge was decreased compared to that prior to the two week challenge (S8 Fig). Since protection correlates with antibody titers, if antibody titers do not wane precipitously after twelve weeks, it is possible that protection could be maintained for much longer than twelve weeks.

The role of antibodies in PyE140 protection was examined in two passive transfer studies in outbred and inbred mice. Although only one recipient mouse in each strain was sterilely protected, there was a significant delay in the onset of parasitemia for the remaining 13 of 14 recipient mice. The lack of sterile protection in the recipient mice was likely due to the relatively low PyE140 antibody titers in the recipient mice, again suggesting that anti-PyE140 antibodies play a pivotal role in protection.

E140 is found in every *Plasmodium* species where genomic sequence is available and is well conserved, with amino acid identity ranging from 34–92% among species. More importantly, it is highly conserved (95–99%) in *P*. *falciparum* strains isolated from different locations around the world (Fig 10A). E140 has no known function or homology with any functional domain that would suggest a potential role or association with any parasite phenotype. Protein structure algorithms predict that the E140 protein has five transmembrane domains, presumably spanning a parasite or host-derived membrane (S2 Fig). PyE140 displays distinct patterns of protein expression in mature sporozoites, late liver, and late schizonts stages by IFA. It traffics to the anterior and posterior ends of the sporozoite, the parasitophorus vacuole (PV) space of the late liver stage and around developing merozoites in the late schizont stage. Other groups have shown that PfE140 is expressed in mature salivary gland sporozoites, as well as oocyst-derived sporozoites and oocysts [36].

Western blot analysis indicates that the PyE140 antigen is processed during erythrocytic schizogony. However, this proteolytic cleavage is distinct from the sporozoite stage, as a predominant band of ~82kDa protein was detected in salivary gland sporozoites. It is unknown if this processing also occurs during intrahepatic schizogony. Several *Plasmodium* proteins are cleaved at different parasite stages where their function and processing play a significant role. This is the case for PfCSP, where site-specific cleavage is critical for hepatocyte invasion [37, 38]. Other proteins intrinsically involved in the invasion of erythrocytes are also subject to proteolytic cleavage, including AMA1 [39–41] and MSP1 [42, 43]. The shedding phenomenon is mediated by an integral membrane subtilisin-like protease, PfSUB2 [44]. PyE140 protective antibodies target blood stage schizonts, the same stage in which PyE140 is proteolytically processed, raising the possibility that the mechanism of PyE140 protection could involve the disruption of the PyE140 protein processing. However, the significance of the PyE140 cleavage in schizonts is currently unknown. It is notable that an independent proteomic profiling study of *P*. *falciparum* designed to identify proteins processed at the end of the blood stage through egress of merozoites did not demonstrate processing of PfE140 [45]. Although PfE140 derived peptides were reproducibly identified from membrane fractions of late trophozoites/early schizonts, as well as from both rupturing and arrested merozoites, all PfE140 peptides were recovered from gel slices of approximately 75–90 kDa, which is consistent with the size of the largest visible band that we observed. It is possible that the discrepancy with our observations of PyE140 could be due to the different methodologies used.

The PfE140 antigen has many of the same features as its *P*. *yoelii* ortholog and merits efforts to evaluate its potential as a malaria vaccine. One important requirement of a malaria vaccine is the ability to induce strain transcending immune responses that can circumvent the antigenic polymorphism present in parasites from endemic regions. PfE140 exhibits a high degree of amino acid conservation (≥95% identity) among twelve laboratory-adapted strains of *P*. *falciparum* originally isolated from geographically diverse regions of the world. An in-depth analysis of the PfE140 variation observed between these twelve lab-adapted strains and circulating isolates revealed that only a minority of the amino acid differences in the lab-adapted strains were identified in the circulating parasite isolates, suggesting that much of the variation in the lab-adapted strains is due to culture adaptation and not from immune pressure. In addition, the frequency and distribution of PfE140 nsSNPs are quite low; only 13 of 791 amino acid positions have a 5% or higher nsSNP frequency in all parasite isolates reported by the MalariaGEN *P*. *falciparum* community project. In general, PfE140 seems to be more conserved than other *P*. *falciparum* vaccine candidates, such as AMA1, CelTOS, MSP1, and SSP2/TRAP, with only CSP and RH5 being either comparable or more conserved than PfE140.

## Conclusions

Immunization targeting the PyE140 antigen induces up to 100% sterilizing protection of mice against either a sporozoite or blood stage challenge. Protection is maintained for at least 12 weeks following immunization of mice and is mediated by persistent antibodies against erythrocytic parasites. Antibodies to PfE140 clone 3D7 recognize parasites from multiple lab-adapted *P*. *falciparum* strains, illustrating its antigenic conservation and potential as a vaccine effective against a wide variety of heterologous parasites. Therefore, the E140 antigen represents a promising malaria vaccine candidate.

## Supporting information

S1 Fig Nucleotide and amino acid sequence of the re-annotated PyE140 gene. (A) Nucleotide sequence of the re-annotated PyE140 gene. Uppercase indicates coding exon sequence and lowercase indicates non-coding intron sequence. (B) Amino acid sequence of the re-annotated PyE140 protein. Due to a PCR-generated error, the DNA-PyE140na and HuAd5-PyE140na vectors express a PyE140 protein with a glycine at position 537, instead of a serine (shown in red). (C) Pairwise alignment of PyE140 and PfE140 protein sequences was performed using ClustalW and displayed using Color Align Conservation: http://www.bioinformatics.org/sms2/color_align_cons.html. Amino acids highlighted in black are identical between PyE140 and PfE140, and those highlighted in gray are similar.
(PDF)

S2 Fig Predicted transmembrane domains within PyE140 and PfE140. (A) PyE140 and (B) PfE140 transmembrane domains were predicted using Protter: wlab.ethz.ch/protter/start/ [46].
(PDF)

S3 Fig Temporal expression of PyE140 in developing liver stage parasites. (A-D) Lack of PyE140 staining 24 hours after infection. Immunofluorescent micrograph of a liver cryosection containing *P*. *yoelii* parasites 24 hours after infection were stained with (A) PyE140 and (B) PyHsp70 antisera. (C) DAPI was used to visualize nuclei. (D) Merge of A, B, and C. PyE140 (red), PyHsp70 (green) and DAPI (blue). Scale bar– 10 μm. (E-H) PyE140 staining 48 hours after infection. Immunofluorescent micrograph of a liver cryosection containing *P*. *yoelii* parasites 48 hours after infection were stained with (E) PyE140 and (F) PyHsp70 antisera. (G) DAPI was used to visualize nuclei. (H) Merge of E, F, and G. PyE140 (red), PyHsp70 (green) and DAPI (blue). Scale bar indicates 10 μm.
(TIF)

S4 Fig Flow cytometry gating strategy for the analysis of PyE140-specific cellular responses of murine lymphocytes in the spleen and liver. (A) Spleen: cells are gated to remove aggregates and to select singlets, viable cells, CD3^+^ T cells, small lymphocytes, and either CD8^+^ or CD4^+^ T cells. (B) Liver: cells are gated by time, to select lymphocytes, remove aggregates, and to select singlets, viable cells, CD3^+^ T cells, small lymphocytes, and either CD8^+^ or CD4^+^ T cells. PyE140-specific T cells were identified by the production of IFN-γ, MIP1α, TNF, or IL-2 following stimulation with either PyE140-A or PyE140-B peptide pools. Memory phenotype was determined by the expression of CD44 and CCR7 on either the total CD8^+^ or CD4^+^ T cell population (density plot) or cells producing IFN-γ (red overlay).
(PDF)

S5 Fig Additional PyE140-specific cellular responses induced by PyE140 immunization. CD1 mice were immunized with DNA and HuAd5 vectors expressing PyE140na or null vectors that do not express a *P*. *yoelii* antigen at weeks 0 and 6. Two weeks after the boost, lymphocytes isolated from spleen and liver were stimulated with peptide pools PyE140-A or PyE140-B for 4 hours for intracellular cytokine staining and subsequent analysis by flow cytometry. Responses are background subtracted using the DMSO negative control stimulations. The frequency of CD8^+^ T cells from spleen producing (A) IL-2, and the frequency of CD4^+^ T cells from spleen producing (B) MIP1α, (C) TNF, and (D) IL-2 are shown. The frequency of CD8^+^ T cells from liver producing (E) MIP1α, (F) TNF, and (G) IL-2 are shown. The frequency of CD4^+^ T cells from liver producing (H) IFN-γ, (I) MIP1α, (J) TNF, and (K) IL-2 are shown. ** indicates *p*<0.01, * indicates *p*<0.05, and NS indicates non-significant by Mann-Whitney.
(PDF)

S6 Fig PyE140-specific T cells do not mediate protection. CD1 mice immunized with DNA and HuAd5 vectors expressing PyE140na or null vectors were depleted of CD4^+^ and/or CD8^+^ T cells and challenged with 300 *P*. *yoelii* sporozoites. Protection was assessed by blood smears and is shown in Fig 5. Shown here are the frequency of lymphocyte subsets in the spleens of two additional mice per group that were euthanized on the day of challenge: frequencies of CD8^+^ T cells by (A) intracellular and (B) surface staining and CD4^+^ T cells by (C) intracellular and (D) surface staining. Filled symbols represent the results from the PyE140-immunized mice and open symbols represent the results from the null-immunized mice. (E) Gating strategy used to identify lymphocyte frequencies in the spleens of T cell depleted mice. Representative examples of non-depleted, depleted, and partially depleted samples are shown.
(PDF)

S7 Fig DNA-PyE140 and HuAd5-PyE140 vectors express PyE140. Western blot showing PyE140 expression by DNA-PyE140na, HuAd5-PyE140 native (HuAd5-PyE140na) and HuAd5-PyE140 codon-optimized (HuAd5-PyE140co) vectors. (A) 293-ORF6 cells were mock infected, transfected with 8 μg of DNA-PyE140na, infected with HuAd5 null, HuAd5-PyE140co or HuAd5-PyE140na at an MOI of 500 pu/cell and harvested 24 hours or 48 hours post-infection/transfection. Lane 1, Marker; Lane 2, Mock (48 hours); Lane 3, DNA-PyE140na (24 hours); Lane 4, HuAd5 null (24 hours); Lane 5, HuAd5 null (48 hours); Lane 6, HuAd5-PyE140co (24 hours); Lane 7, HuAd5-PyE140co (48 hours); Lane 8, HuAd5-PyE140na (24 hours), and Lane 9, HuAd5-PyE140na (48 hours). (B) 293-ORF6 cells were mock infected, transfected with 8 μg of DNA-PyE140na, infected with HuAd5 null, HuAd5-PyE140co or HuAd5-PyE140na and harvested 24 hours post-infection/transfection. Lane 1, Marker; Lane 2, Mock; Lane 3, DNA-PyE140na; Lane 4, HuAd5 null (MOI = 500); Lane 5, HuAd5-PyE140na (MOI = 500); Lane 6, HuAd5-PyE140na (MOI = 6,500); Lane 7, HuAd5-PyE140co (MOI = 500). For both Westerns, the primary antibody was sera from CD1 mice immunized with DNA and HuAd5 vectors expressing PyE140na and the secondary antibody was goat anti-mouse IgG conjugated to alkaline phosphatase. Signals were visualized with the KPL BCIP/NBT phosphate substrate system.
(TIF)

S8 Fig PyE140 antibody titers at one and eleven weeks post-immunization using vectors expressing PyE140na and PyE140co. (A-D) The same data shown in Fig 6 are shown here to provide a comparison between antibody titers elicited by vectors expressing PyE140na or PyE140co. CD1 mice were immunized with a single dose of HuAd5-PyE140na or HuAd5-PyE140co, or with a prime-boost regimen consisting of a DNA-PyE140na prime and a HuAd5-PyE140na or HuAd5-PyE140co boost. A group of mice immunized with DNA and HuAd5 null vectors and a group of naïve mice were also included in the study. Black indicates responses induced by HuAd5-PyE140na and gray indicates responses induced by HuAd5-PyE140co. (A) Anti-Py sporozoite IFA titers of mice 1 week after the last immunization (1 week before challenge). (B) Anti-Py sporozoite IFA titers of mice eleven weeks post-boost (1 week before challenge). (C) Anti-Py blood stage IFA titers of mice 1 week after the last immunization (1 week before challenge). (D) Anti-Py blood stage IFA titers of mice eleven weeks post-boost (1 week before challenge). (E-H) The same data shown in Panels A-D are shown to provide a comparison between antibody titers at one and eleven weeks post-immunization. Black indicates responses at one week post-immunization and gray indicates responses at eleven weeks post-immunization. (E) Anti-Py sporozoite IFA titers elicited by immunization with a DNA-PyE140na prime and a HuAd5-PyE140na boost at one and eleven weeks post-boost. (F) Anti-Py sporozoite IFA titers elicited by immunization with a DNA-PyE140na prime and a HuAd5-PyE140co boost at one and eleven weeks post-boost. (G) Anti-Py blood stage IFA titers elicited by immunization with a DNA-PyE140na prime and a HuAd5-PyE140na boost at one and eleven weeks post-boost. (H) Anti-Py blood stage IFA titers elicited by immunization with a DNA-PyE140na prime and a HuAd5-PyE140co boost at one and eleven weeks post-boost. ****, ***, **, *, and NS indicate *p*<0.0001, *p*<0.001, *p*<0.01, *p*<0.05, and not significant by Mann-Whitney.
(TIF)

S9 Fig PyE140 immunization does not reduce liver stage parasite burden. 11 week old CD1 mice were immunized with DNA vaccines encoding PyE140 or PyCSP at week 0 and HuAd5 vaccines encoding PyE140co or PyCSP at week 6. Groups of null-immunized and naïve mice were also included. Two weeks after the boost, mice were challenged with 5,000 *P*. *yoelii* 17XNL sporozoites by subcutaneous injection. Livers were harvested 42 hours after challenge for evaluation of Py18S mRNA copies/murine GAPDH mRNA copies by RT-qPCR. (A) Filled symbols represent detectable Py18S copies and open symbols represent Py18S copies below the limit of detection (10 copies of Py18S RNA) and are plotted using ½ the limit of detection (5 copies of Py18S mRNA). ** indicates *p*<0.01 and NS indicates not significant by Mann-Whitney. Standard curves for each run demonstrate reproducible quantification of (B) Py18S and (C) murine GAPDH mRNA. Data represent standard curves from eight plates run in replicates of two (n = 16 per standard dilution). Two outlier data points from the GAPDH standard curve were omitted from the total data analysis. Unique symbols identify data from each plate.
(TIF)

S10 Fig PfE140 expression in *P*. *falciparum* schizonts by IFA. PfE140 expression in schizonts was detected by IFA. Sera from mice immunized against PfE140 were used to identify PfE140 expression in blood stage schizonts. Green staining indicates PfE140. PfE140 is expressed in individual merozoites in late stage (A) 3D7 strain and (B) 7G8 strain schizonts. Scale bar indicates 10 μm.
(TIF)

S1 Raw Images (PDF)
