# Supplementary material for: Novel malaria antigen Plasmodium yoelii E140 induces antibody-mediated sterile protection in mice against malaria challenge
Source: PLoS One. 2020 May 14;15(5):e0232234. doi: 10.1371/journal.pone.0232234 (PMC7224506; doi:10.1371/journal.pone.0232234)
Supplement: S1 Fig — (A) Nucleotide sequence of the re-annotated PyE140 gene. Uppercase indicates coding exon sequence and lowercase indicates non-coding intron sequence. (B) Amino acid sequence of the re-annotated PyE140 protein. Due to a PCR-generated error, the DNA-PyE140na and HuAd5-PyE140na vectors express a PyE140 protein with a glycine at position 537, instead of a serine (shown in red). (C) Pairwise alignment of PyE140 and PfE140 protein sequences was performed using ClustalW and displayed using Color Align Conservation: http://www.bioinformatics.org/sms2/color_align_cons.html. Amino acids highlighted in black are identical between PyE140 and PfE140, and those highlighted in gray are similar. (PDF) [file pone.0232234.s001.pdf]

Fig S1A. PyE140 nucleotide sequence.

ATGGGAGACGTTGACAATGTGTTAATAAGTATCAAAAAAATAGAATCAATAAAAAGCCAATTAAACCAGT
TAAACAAAATTATACAAAATGAATTTGGGTCTTATTGTGGGCGAAAAAATAGAAGTATAAATCTTGAAAT
ACATCATAATGAATTTGATAAAAGTATATTCAAACGTTTATATTCATCATGGAGAATGGAAGATCTTAAT
AATTTTAACGGGAAAAGTGTTATAAAAATAATGGAAAGAAATCCATATGTTATATTTTTTTTTTTTTTTA
TAATGATTTTTATTATTGTTTATTTAATTTCATTTATTTTGTATACTAAATGGTTTAAAAAATTATTAAA
AAAATTTTCGAATTCACACAAAAATAATAAAGATAAAGAAGAAGATTGGGTAAAAAAAAATAAAGCTTAT
AGAAATTCTAATAGCACACATGGTACTATTAATAAGGATAATTATAATCAGGAACTTGATGAGCTTCATA
ATAGTGATGAAAATGAAGAAAATAGTAATGTTATAAATATTGTAAAAAAGAGAGCTTATAATTTAGTAAT
TAATTTGATAGTTTGTTCTTTTCTTATTTGTCTTATTTTTTTGGGAATTTGGACAATATTTATTTTTGCA
GATACACAAAAAGGAATTAATATGAATATCTGTGGATTATCAAAAACAGTTGAACAGTTTCTTATTGATA
AATGCCCCGACACAAAAAATGTAAATCCACAATGTTATTCTTTAGAGCATGTTATTAATGATGCTGTTTC
AGTAATGAATCAGTATCAACTCACCAAAGAATTTGTTAAAAATAAAACGAATTTGAACAAGAATAAGGGC
TTGCCTATTGTTTTAAgtaggtttatatcactacccgcgtttctcctttgcacctaaacaagttgcgtct
gtttttgcatctaaaatttgcggctgctccttcacatctaaaaatgttgcgtctgcttttgcgtctgctt
ttgcgtctgcttttgcatctgccccttcacatctatttctttttttccccgtatagAGTACCAAACCGGAT
TCAACATGCTAGCAAAACTTAGAGACAACATAGATAAAAATGTTAAAAAATTAGAGAACGGATATCTTCA
CACATATCCAGTTTTAACAAAACTTAGATTTACTTTGGATGAAATTGTTTCGAAGGGAGAGAATCTATTA
AATCAGGCTGAATCTATTATTGATTCTTCAAAAGAAGAAATTGGAAAAATATTCAATAATGTAGATAATG
CTATAGCTAATACTGTACATAATAATGTTCCGTCTTTATCATCAAAAATAAGTGGATTAGGAATATATAT
AAAAAAACAAGACGAAAATTTAAAAATACGATATATATTAAATAAATTTACAGTTACAATGATAATTTTA
AGTATAGTCATATTATTATTTTCATTACTTGTGTTAATAGGAATGTTATCTTATATGTATTTTTTAATAA
GAGGCCATTCAATAAATGAAAAATTTTTTTCAAAATTACTTGGTTTTTTTAGTGGAACATTTGGATTTTT
AGCAATTATAATTTTAATAATAGGTACGGCATTATTAAGTTTATCTGTTTTGGGTGGAACAAGTTGTATT
ATATCTGATCGAATATTAAAAAATGAATTTACTTTTGATTTTTTAAGCGAAAATAAAATTGGTTATTGTT
TACAGAATCCAGATGAATCTATTATTAATAAAAATATTGTAAAAAAATATGCAAACACTCTTGACTCTTT
AAATACAAATGATATATATAATAGTGTTGAAGGCTATAGTGGTTATTTTGATAAAATTAAGGATGAATAT
AAACAACATTCTAAAATTATAAATGAAAATATGTGGATAATTATTCCTACAGATAATAATAAATATGTAA
AAAATGTTAAATCAGATATTATTAAAAAATCATTATTAGGAACATGTTTAACAAAAGAAAGTGCCCAATT
TGAAGAGTATCATCTTATGGGAACAGATGCTTATATGAAATATATAAATAAATTTGGTTTGCTAAATAAT
TATGAGATGTGTTTTGAAGACCCATCATGTGAAAATAACGACAGAAAATACAATATCAATTATAACTCTA
AAGTTACAGACCCAAAATATCTTGATGTTAAACGTAATAGAGTCATGCTTTATCAAGATAGCGATTTTGA
TAATGTACTTGAAGTGTTCATATTAAAATCAAAAATTAATAATGATAAAATTTTTAATATTAGCGATTTA
GATGAAACAAAGAAGGAAAATATAACATGGAGAGAATATACACCAAAAAATGGAGCCGGAGAAAATAAAA
AATCTATTGTTCAAACATATTTTGAGAAAGCTATTGAATATATGAAATTTGAAAATGTTTTAACTTTACT
TAAAGAAGTTAATAATCATATAAATTCATTTAAAAATGTTATTATTGAAAAAGCTAATTCATTAGTAGAT
AATACAAATTGTAGTAGATTTATTAATGTACTAACTAATATAAGACATAATTATTGTGACAATGGAATTT
TAAAATTAACTCGATTATCAGTCATACTTATTTCATGTGGATTTGTTTCCTTTTGTCTTTGGTACCTTTT
CCTTTTTTTTTGGATATACCATCAAATGAAGATTATTTGA

Fig S1B. PyE140 amino acid sequence.

```
MGDVDNVLISIKKIESIKSQLNQLNKIIQNEFGSYCGRKNRSINLEIHHNEFDKSIFKRLYSSWRMEDLN
NFNGKSVIKIMERNPYVIFFFFFIMIFIIVYLISFILYTKWFKKLLKKFSNSHKNNKDKEEDWVKKNKAY
RNSNSTHGTINKDNYNQELDELHNSDENEENSNVINIVKKRAYNLVINLIVCSFLICLIFLGIWTIFIFA
DTQKGINMNICGLSKTVEQFLIDKCPDTKNVNPQCYSLEHVINDAVSVMNQYQLTKEFVKNKTNLNKNKG
LPIVLKYQTGFNMLAKLRDNIDKNVKKLENGYLHTYPVLTKLRFTLDEIVSKGENLLNQAESIIDSSKEE
IGKIFNNVDNAIANTVHNNVPSLSSKISGLGIYIKKQDENLKIRYILNKFTVTMIILSIVILLFSLLVLI
GMLSYMYFLIRGHSINEKFFSKLLGFFSGTFGFLAIIILIIGTALLSLSVLGGTSCIISDRILKNEFTFD
FLSENKIGYCLQNPDESIINKNIVKKYANTLDSLNTNDIYNSVEGYSGYFDKIKDEYKQHSKIINENMWI
IIPTDNNKYVKNVKSDIIKKSLLGTCLTKESAQFEEYHLMGTDAYMKYINKFGLLNNYEMCFEDPSCENN
DRKYNINYNSKVTDPKYLDVKRNRVMLYQDSDFDNVLEVFILKSKINNDKIFNISDLDETKKENITWREY
TPKNGAGENKKSIVQTYFEKAIEYMKFENVLTLLKEVNNHINSFKNVIIEKANSLVDNTNCSRFINVLTN
IRHNYCDNGILKLTRLSVILISCGFVSFCLWYLFLFFWIYHQMKII
```

Fig S1C. Alignment of PyE140 and PfE140 amino acid sequences.

```
PyE140 MGDVDNVLISIKKIESIKSQLNQLNKIIQNEFGSYCGRKNRSINLEIHHN------EFDKSIFKRLYSSWRMEDLNNFNG 74
PfE140 MVDFNDLSVELKKTELIKEDLRNLSHIINNEFSYFCQNENKNVSFNNNISSYYNDDIFSKSTLNNLYTSWKLEDFSHFDF 80

PyE140 KSVIKIMERNPYVIFFFFFIMIFIIVYLISFILYTKWFKKLLKKFSNSHKNNKDKEEDWVKKNKAYRNSNSTHGTINKDN 154
PfE140 SSILDILKRNQYVMCSIYFLLIFSCIYFLTLLLYTKCIKTTLKKWFCRYCSEN----------------------INEN- 137

PyE140 YNQELDELHNSDENEENSNVINIVKKRAYNLVINLIVCSFLICLIFLGIWTIFIFADTQKGINMNICGLSKTVEQFLIDK 234
PfE140 ---------NSNHNEQRTVLQNVINKSCYFITYSSIIC-LLLFLLLSGITYMHYFIKTKKGIHSNICNIYTRLDKFLLNK 207

PyE140 CPDTKNVNPQCYSLEHVINDAVSVMNQYQLTKEFVKNKTNLNKNKGLPIVLKYQTGFNMLAKLRDNIDKNVKKLENGYLH 314
PfE140 CLDPKKVDTSCYSAEHILNDLSSILEEYKKVKQQAKDDTLLDENTPFPLLERYITTFNKLNVLKNNINKNNTTLENEYFH 287

PyE140 TYPVLTKLRFTLDEIVSKGENLLNQAESIIDSSKEEIGKIFNNVDNAIANTVHNNVPSLSSKISGLGIYIKKQDENLKIR 394
PfE140 TYPALKGISETLTTIISEGNKNFGNARNVIKEVKSTIKYSFHTVDETIRNVFKDSVPKITGLITQAGKSIKGINNKYKIK 367

PyE140 YILNKFTVTMIILSIVILLFSLLVLIGMLSYMYFLIRGHS-INEKFFSKLLGFFSGTFGFLAIIILIIGTALLSLSVLGG 473
PfE140 ERIPKYTNIILLTNIILLLPPFLILLGIIIFMIFILMGYIQKNNNFFIKLFGHFSAYFGLLTIIILSFGILFLSTSVIGG 447

PyE140 TSCIISDRILKNEFTFDFLSENKIGYCLQNPDESIINKNIVKKYANTLDSLNTNDIYNSVEGYSGYFDKIKDEYKQHSKI 553
PfE140 TSCILSERILKNELRFDILNNTLIDYCIKNESAPLIDDDITTSFVAKINSFDTGHIDHNINEYEKHETILKESFFHKSLK 527

PyE140 INENMWIIIP-TDNNKYVKNVKSDIIKKSLLGTCLTKESAQFEEYHLMGTDAYMKYINKFGLL-NNYEMCFEDPSCENND 631
PfE140 FMDYIWIVIMKRENNTFLNRIRTEQVKKSLLITGIINENIKYENMEAIGIRSYLTTLNKIIFPENNGKICFNDIICEKEN 607

PyE140 RKYNINYNSKVTDPKYLDVKRNRVMLYQDSDFDNVLEVFILKSKINNDKIFNISDLDETKKENITWREYTPKNGAGENKK 711
PfE140 NTYNITENSKTTDQKYRNIR-DGMDEHLRNDLDAIVQLFVYKARILKENIFDINDLDSNEKNKIGWSEYTPRNINGTQKK 686

PyE140 SIVQTYFEKAIEYMKFENVLTLLKEVNNHINSFKNVIIEKANSLVDNTNCSRFINVLTNIRHNYCDNGILKLTRLSVILI 791
PfE140 SIINTFLVNVIESINFSEIINFFDKMRDQFNVLKDLILLKIDTLTENTKCNKLVKELINVRKDYCNNVVLNLSTLSVYLI 766

PyE140 SCGFVSFCLWYLFLFFWIYHQMKII 816
PfE140 IFSITSFLLWYLFLFLWFYYNIKPS 791
```
