# Supplementary figures and images for: Novel malaria antigen Plasmodium yoelii E140 induces antibody-mediated sterile protection in mice against malaria challenge
Source: PLoS One. 2020 May 14;15(5):e0232234. doi: 10.1371/journal.pone.0232234 (PMC7224506; doi:10.1371/journal.pone.0232234)

Fig S2A. PyE140 predicted transmembrane domains.

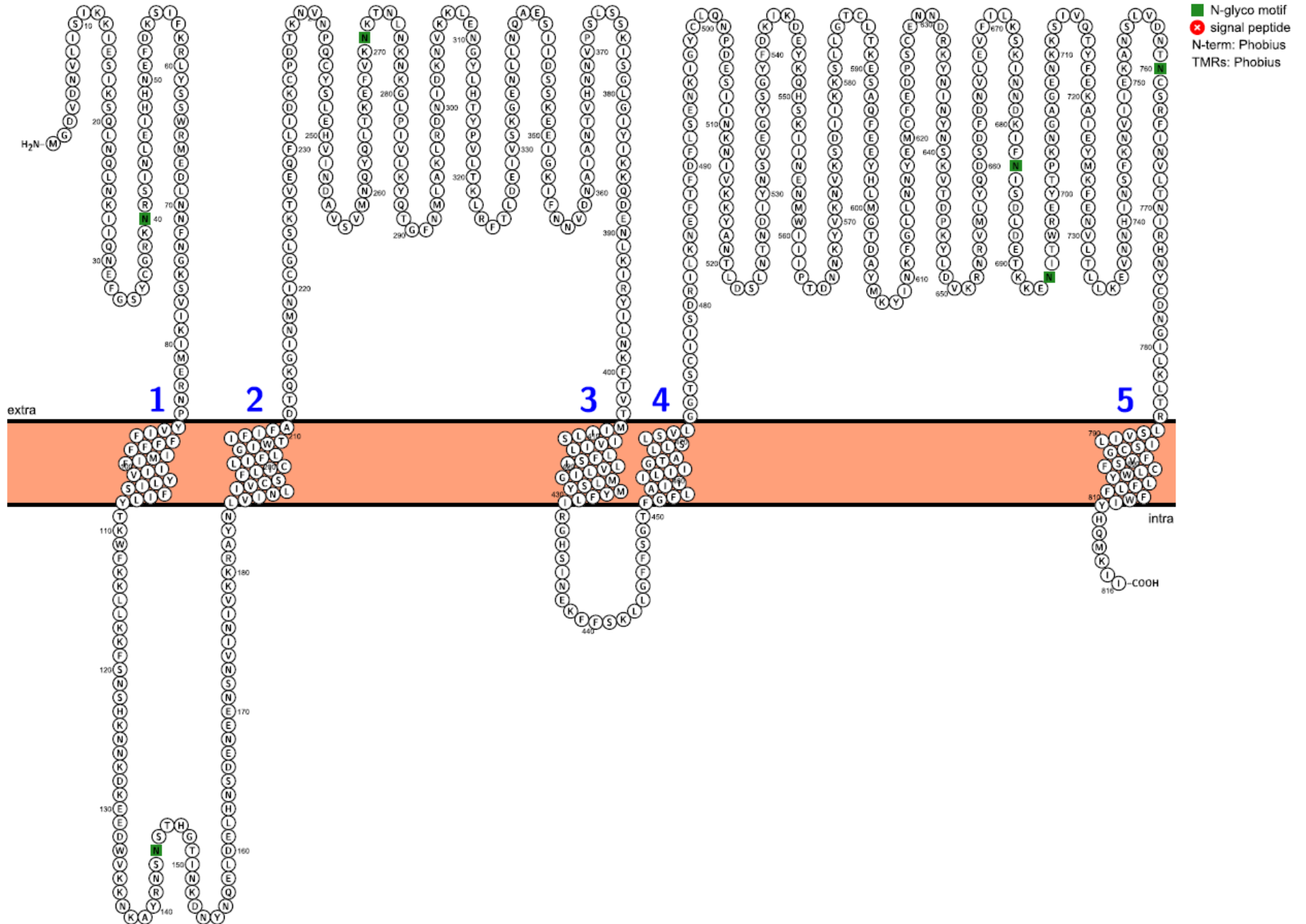

Fig S2B. PfE140 predicted transmembrane domains.

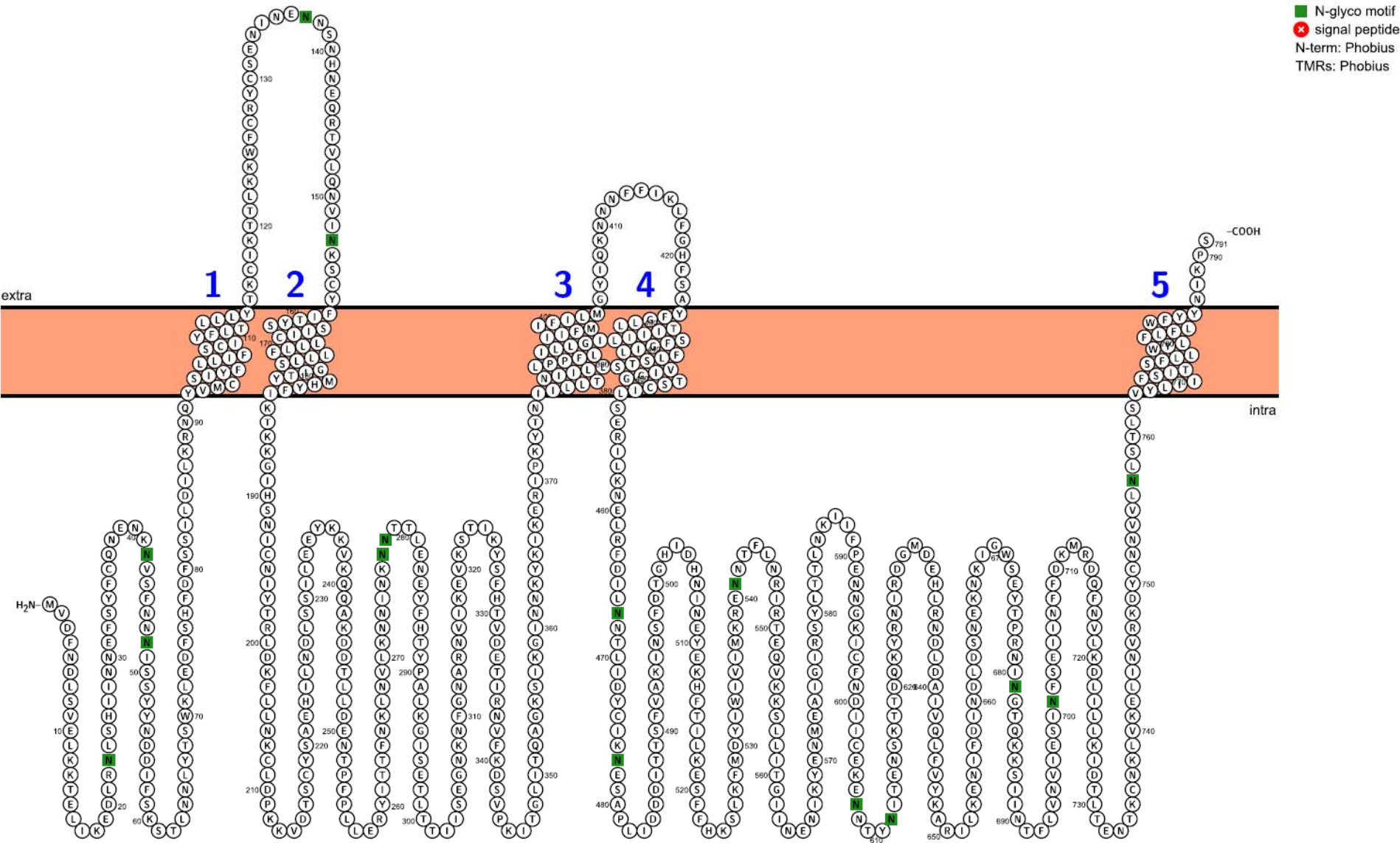

Supplement: S2 Fig — (A) PyE140 and (B) PfE140 transmembrane domains were predicted using Protter: wlab.ethz.ch/protter/start/ [46]. (PDF) [file pone.0232234.s002.pdf]

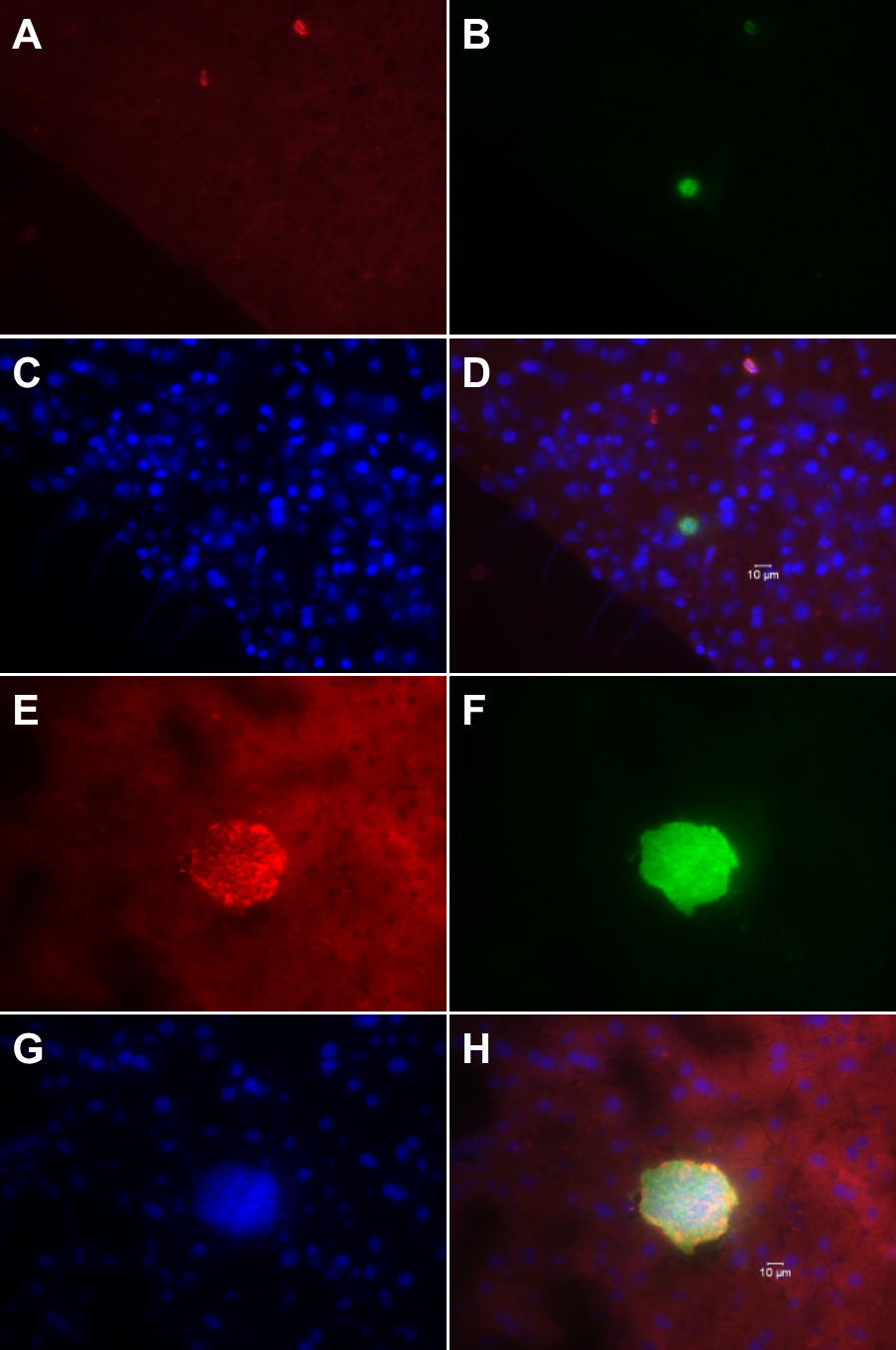

Supplement: S3 Fig — (A-D) Lack of PyE140 staining 24 hours after infection. Immunofluorescent micrograph of a liver cryosection containing P. yoelii parasites 24 hours after infection were stained with (A) PyE140 and (B) PyHsp70 antisera. (C) DAPI was used to visualize nuclei. (D) Merge of A, B, and C. PyE140 (red), PyHsp70 (green) and DAPI (blue). Scale bar– 10 μm. (E-H) PyE140 staining 48 hours after infection. Immunofluorescent micrograph of a liver cryosection containing P. yoelii parasites 48 hours after infection were stained with (E) PyE140 and (F) PyHsp70 antisera. (G) DAPI was used to visualize nuclei. (H) Merge of E, F, and G. PyE140 (red), PyHsp70 (green) and DAPI (blue). Scale bar indicates 10 μm. (TIF) [file pone.0232234.s003.tif]

## A Gating Strategy: Spleen

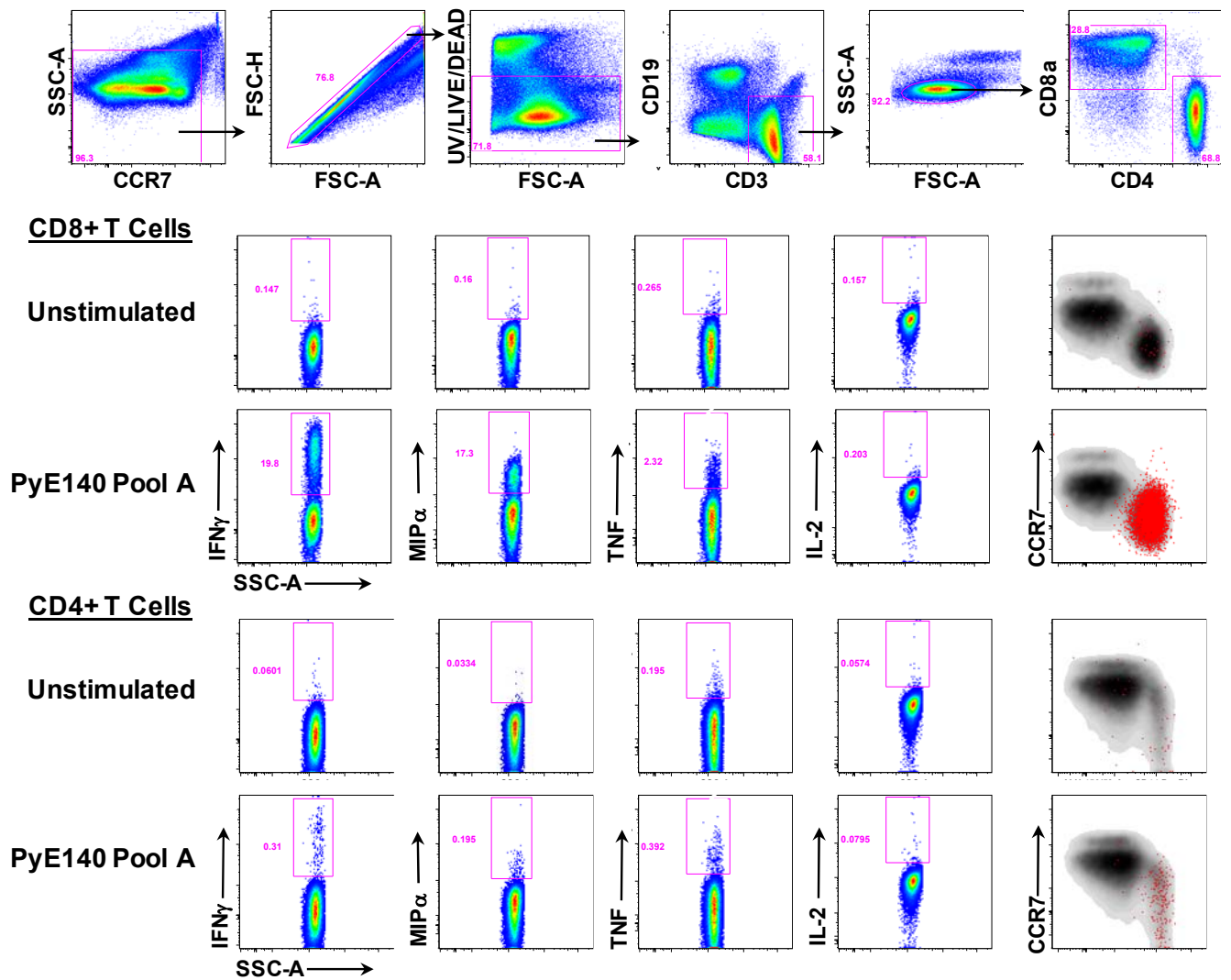

## B Gating Strategy: Liver

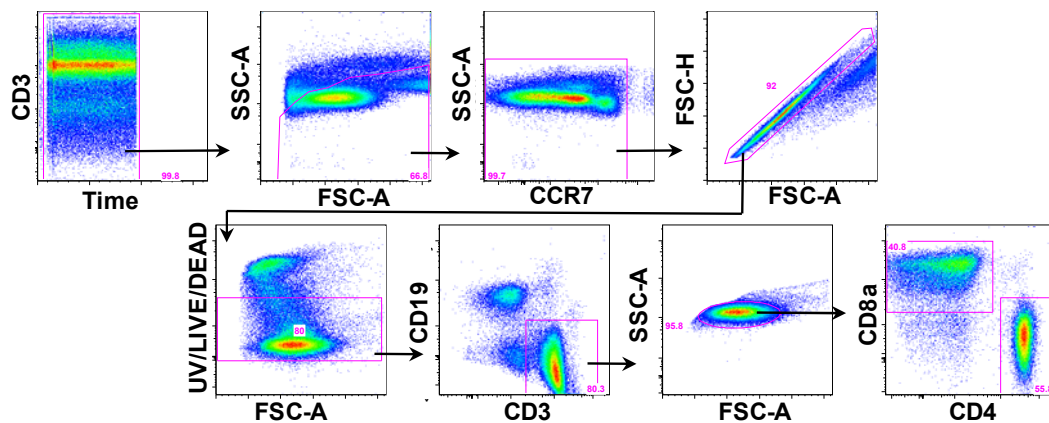

### CD8+ T Cells

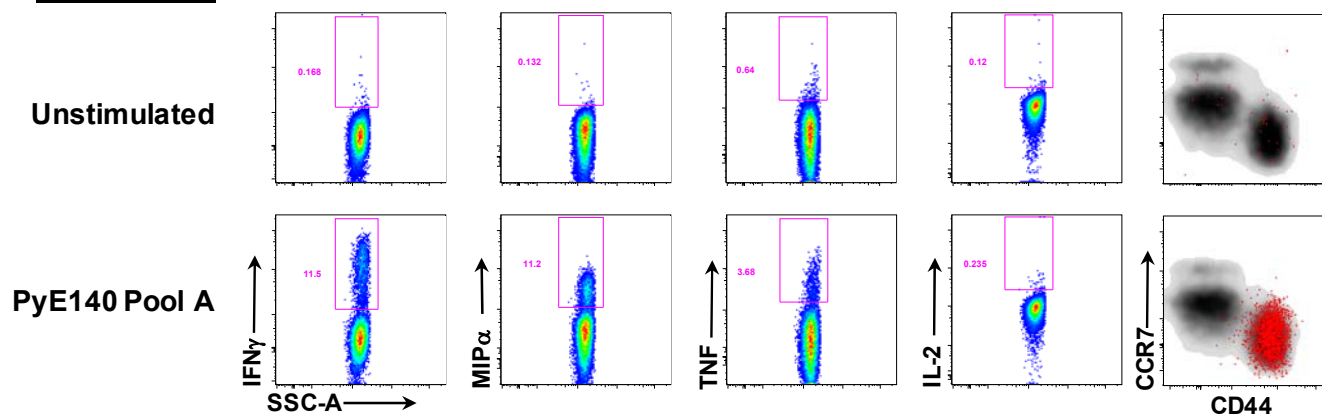

### CD4+ T Cells

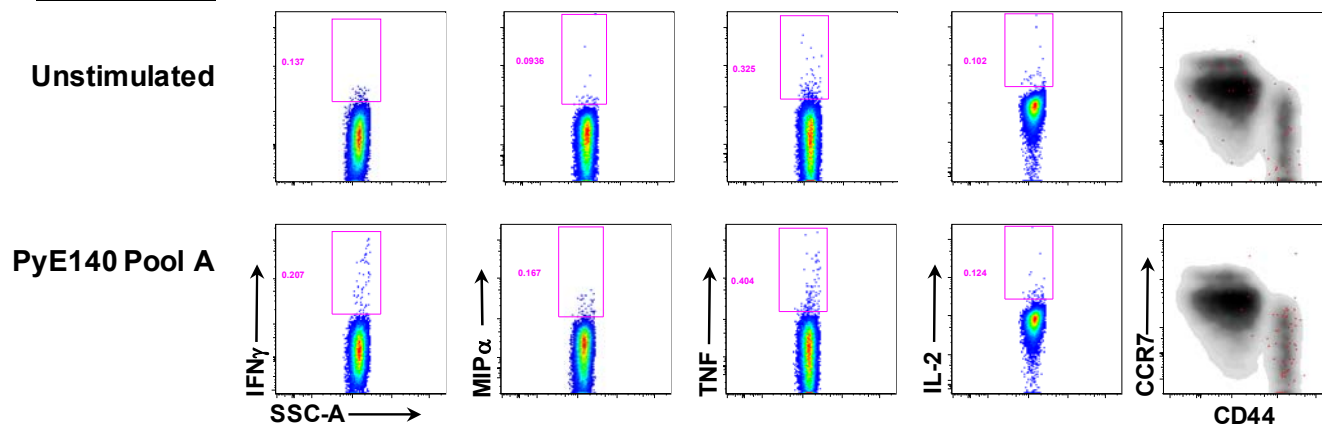

Supplement: S4 Fig — (A) Spleen: cells are gated to remove aggregates and to select singlets, viable cells, CD3+ T cells, small lymphocytes, and either CD8+ or CD4+ T cells. (B) Liver: cells are gated by time, to select lymphocytes, remove aggregates, and to select singlets, viable cells, CD3+ T cells, small lymphocytes, and either CD8+ or CD4+ T cells. PyE140-specific T cells were identified by the production of IFN-γ, MIP1α, TNF, or IL-2 following stimulation with either PyE140-A or PyE140-B peptide pools. Memory phenotype was determined by the expression of CD44 and CCR7 on either the total CD8+ or CD4+ T cell population (density plot) or cells producing IFN-γ (red overlay). (PDF) [file pone.0232234.s004.pdf]

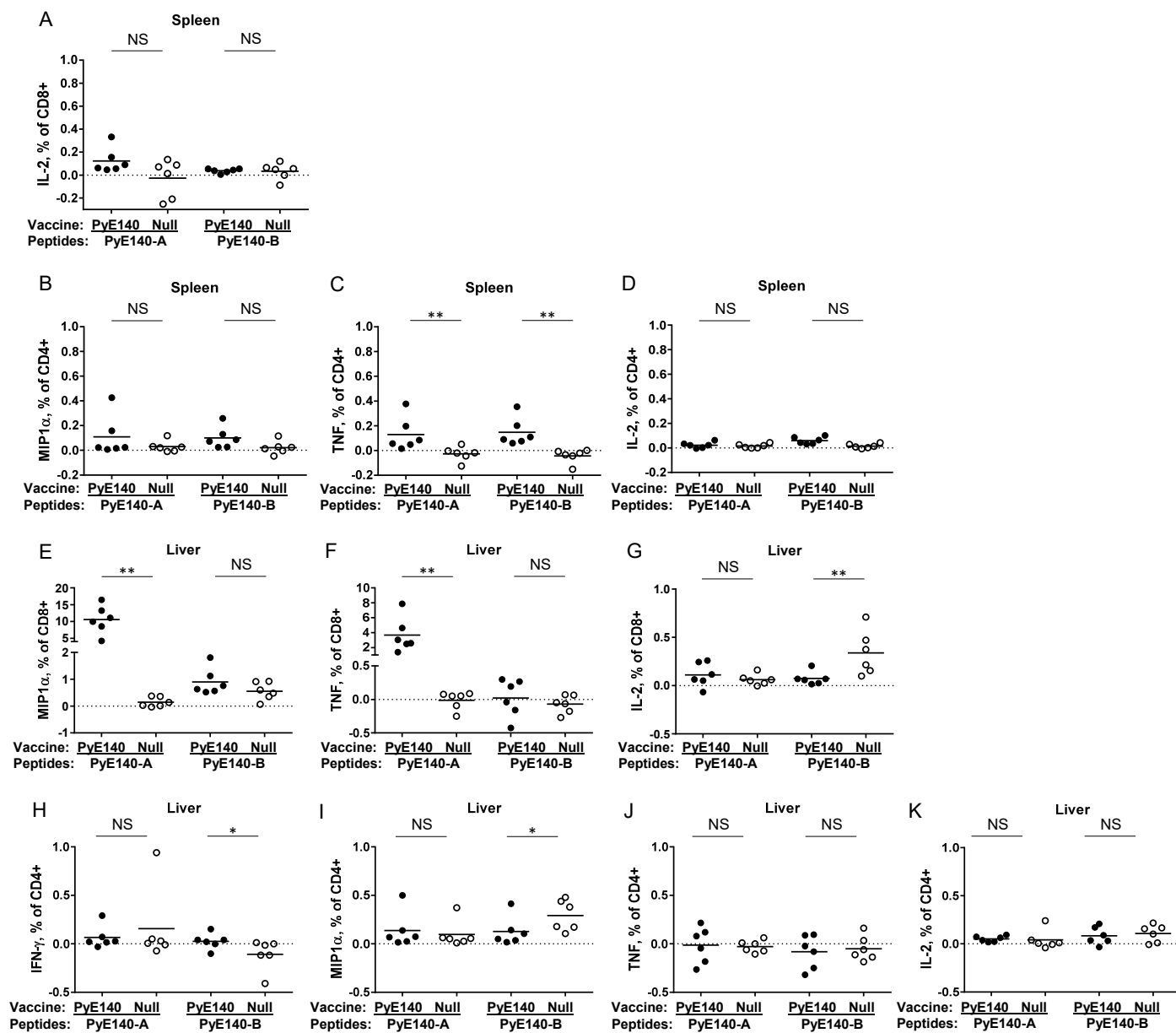

Supplement: S5 Fig — CD1 mice were immunized with DNA and HuAd5 vectors expressing PyE140na or null vectors that do not express a P. yoelii antigen at weeks 0 and 6. Two weeks after the boost, lymphocytes isolated from spleen and liver were stimulated with peptide pools PyE140-A or PyE140-B for 4 hours for intracellular cytokine staining and subsequent analysis by flow cytometry. Responses are background subtracted using the DMSO negative control stimulations. The frequency of CD8+ T cells from spleen producing (A) IL-2, and the frequency of CD4+ T cells from spleen producing (B) MIP1α, (C) TNF, and (D) IL-2 are shown. The frequency of CD8+ T cells from liver producing (E) MIP1α, (F) TNF, and (G) IL-2 are shown. The frequency of CD4+ T cells from liver producing (H) IFN-γ, (I) MIP1α, (J) TNF, and (K) IL-2 are shown. ** indicates p<0.01, * indicates p<0.05, and NS indicates non-significant by Mann-Whitney. (PDF) [file pone.0232234.s005.pdf]

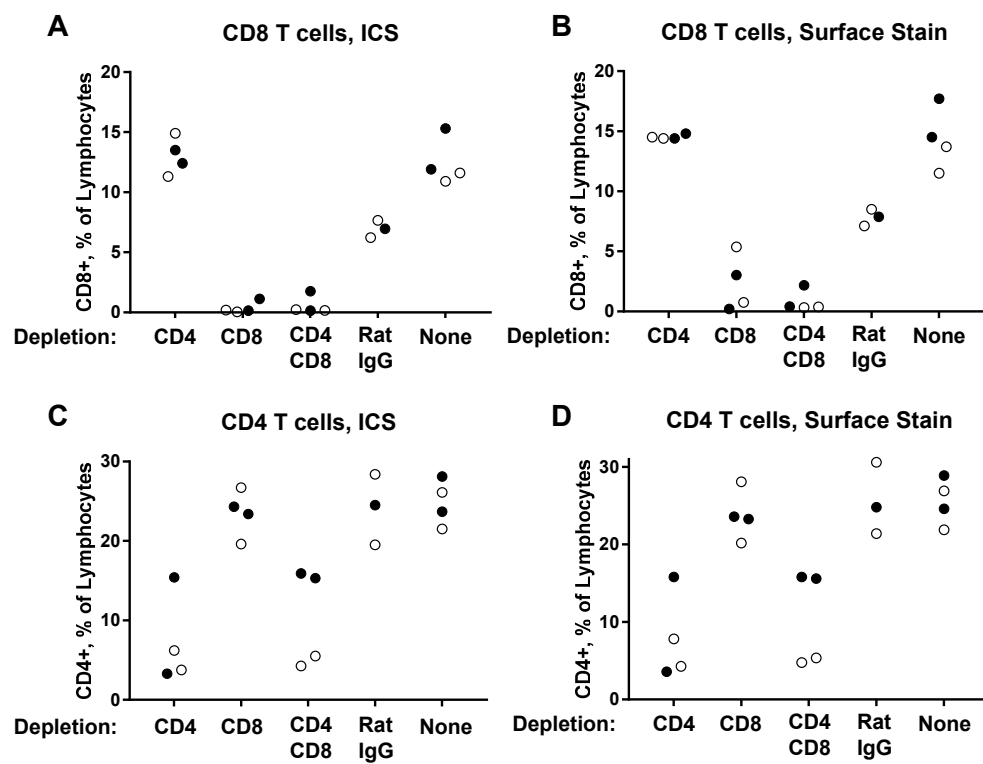

**E** Depletion Gating Strategy

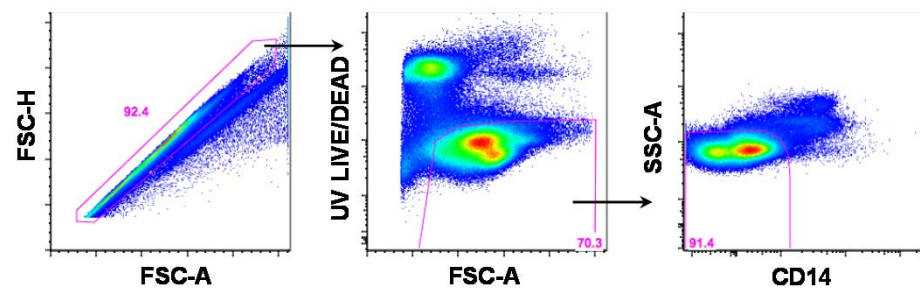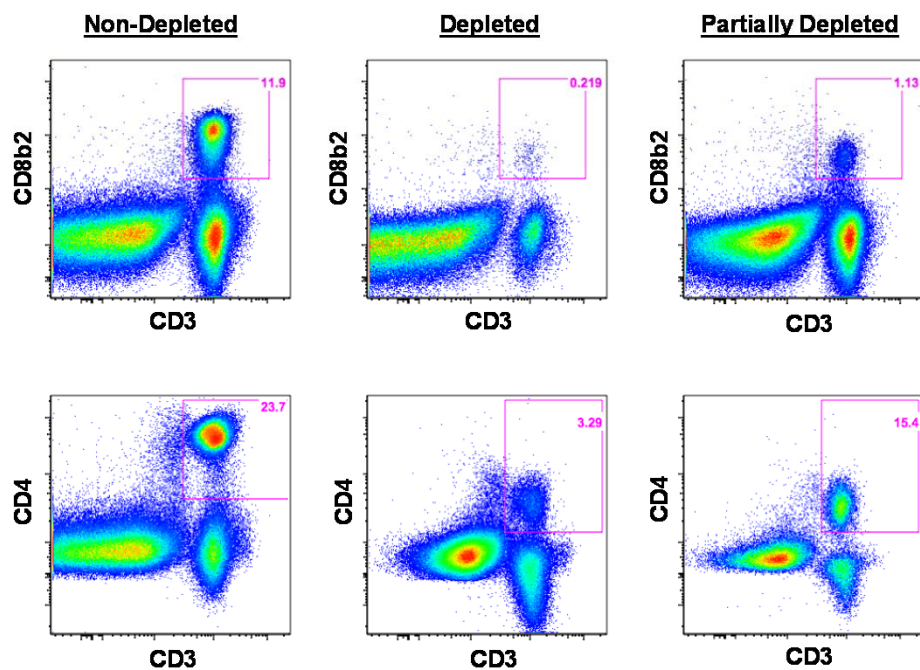

Supplement: S6 Fig — CD1 mice immunized with DNA and HuAd5 vectors expressing PyE140na or null vectors were depleted of CD4+ and/or CD8+ T cells and challenged with 300 P. yoelii sporozoites. Protection was assessed by blood smears and is shown in Fig 5. Shown here are the frequency of lymphocyte subsets in the spleens of two additional mice per group that were euthanized on the day of challenge: frequencies of CD8+ T cells by (A) intracellular and (B) surface staining and CD4+ T cells by (C) intracellular and (D) surface staining. Filled symbols represent the results from the PyE140-immunized mice and open symbols represent the results from the null-immunized mice. (E) Gating strategy used to identify lymphocyte frequencies in the spleens of T cell depleted mice. Representative examples of non-depleted, depleted, and partially depleted samples are shown. (PDF) [file pone.0232234.s006.pdf]

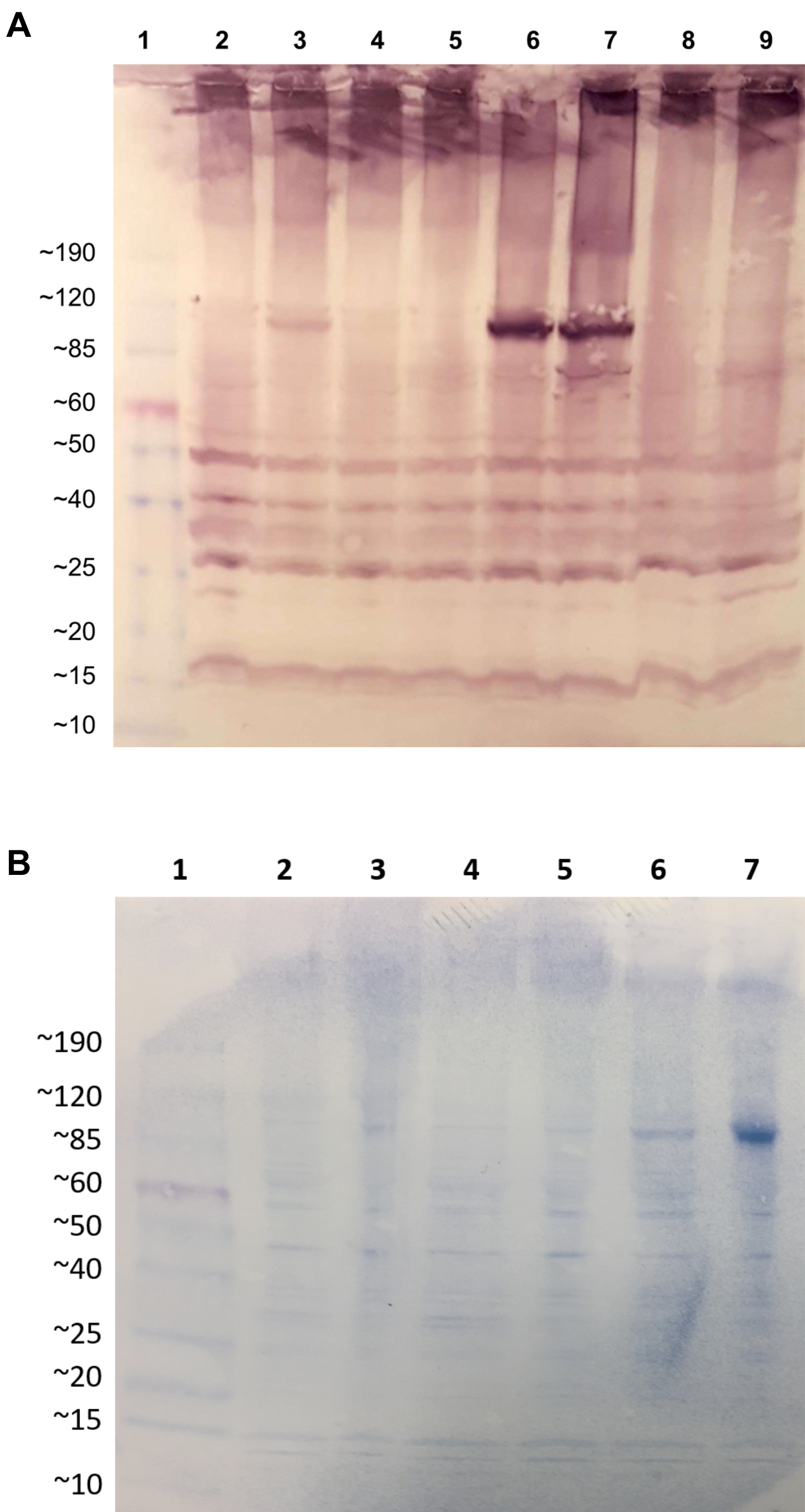

Supplement: S7 Fig — Western blot showing PyE140 expression by DNA-PyE140na, HuAd5-PyE140 native (HuAd5-PyE140na) and HuAd5-PyE140 codon-optimized (HuAd5-PyE140co) vectors. (A) 293-ORF6 cells were mock infected, transfected with 8 μg of DNA-PyE140na, infected with HuAd5 null, HuAd5-PyE140co or HuAd5-PyE140na at an MOI of 500 pu/cell and harvested 24 hours or 48 hours post-infection/transfection. Lane 1, Marker; Lane 2, Mock (48 hours); Lane 3, DNA-PyE140na (24 hours); Lane 4, HuAd5 null (24 hours); Lane 5, HuAd5 null (48 hours); Lane 6, HuAd5-PyE140co (24 hours); Lane 7, HuAd5-PyE140co (48 hours); Lane 8, HuAd5-PyE140na (24 hours), and Lane 9, HuAd5-PyE140na (48 hours). (B) 293-ORF6 cells were mock infected, transfected with 8 μg of DNA-PyE140na, infected with HuAd5 null, HuAd5-PyE140co or HuAd5-PyE140na and harvested 24 hours post-infection/transfection. Lane 1, Marker; Lane 2, Mock; Lane 3, DNA-PyE140na; Lane 4, HuAd5 null (MOI = 500); Lane 5, HuAd5-PyE140na (MOI = 500); Lane 6, HuAd5-PyE140na (MOI = 6,500); Lane 7, HuAd5-PyE140co (MOI = 500). For both Westerns, the primary antibody was sera from CD1 mice immunized with DNA and HuAd5 vectors expressing PyE140na and the secondary antibody was goat anti-mouse IgG conjugated to alkaline phosphatase. Signals were visualized with the KPL BCIP/NBT phosphate substrate system. (TIF) [file pone.0232234.s007.tif]

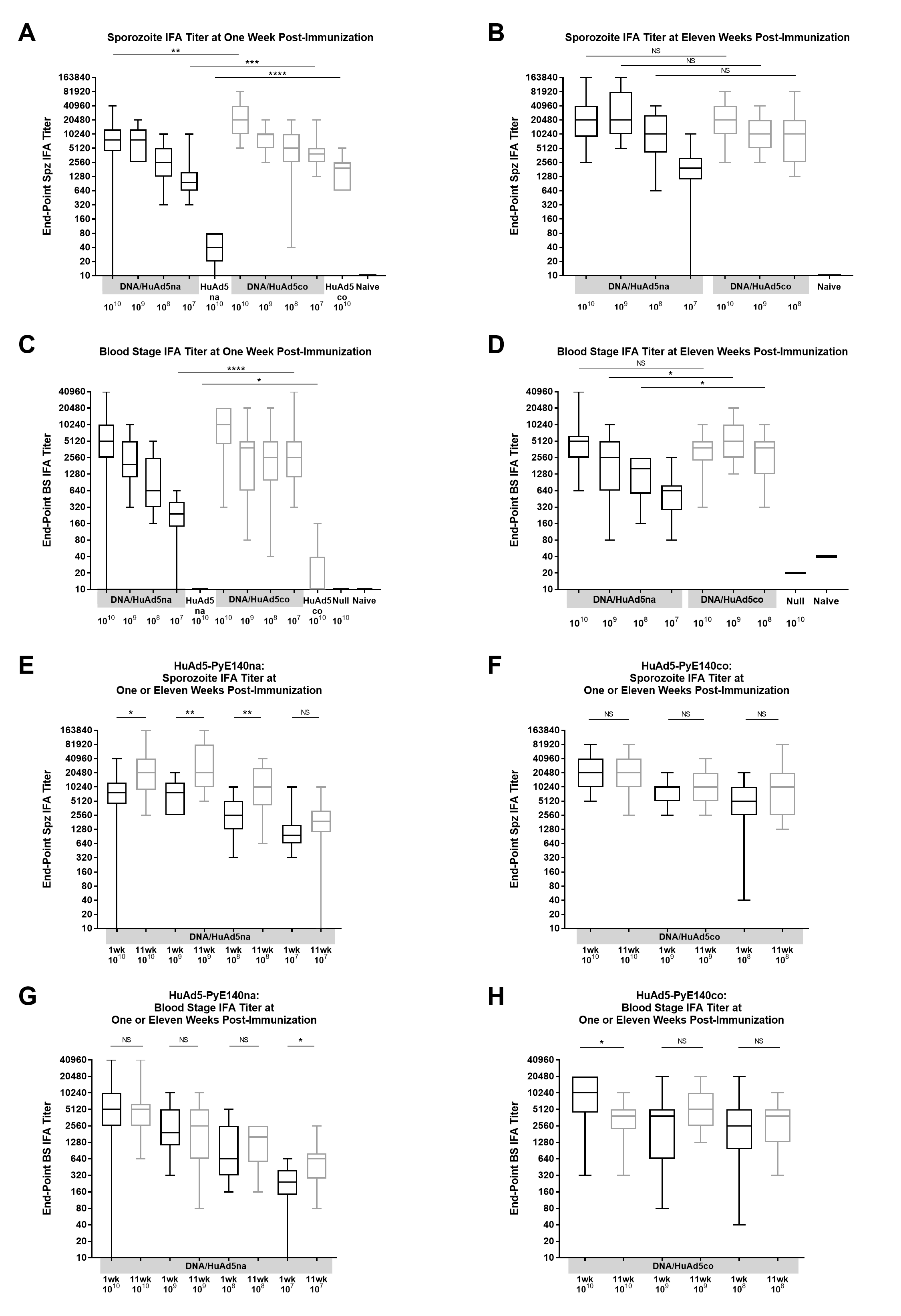

Supplement: S8 Fig — (A-D) The same data shown in Fig 6 are shown here to provide a comparison between antibody titers elicited by vectors expressing PyE140na or PyE140co. CD1 mice were immunized with a single dose of HuAd5-PyE140na or HuAd5-PyE140co, or with a prime-boost regimen consisting of a DNA-PyE140na prime and a HuAd5-PyE140na or HuAd5-PyE140co boost. A group of mice immunized with DNA and HuAd5 null vectors and a group of naïve mice were also included in the study. Black indicates responses induced by HuAd5-PyE140na and gray indicates responses induced by HuAd5-PyE140co. (A) Anti-Py sporozoite IFA titers of mice 1 week after the last immunization (1 week before challenge). (B) Anti-Py sporozoite IFA titers of mice eleven weeks post-boost (1 week before challenge). (C) Anti-Py blood stage IFA titers of mice 1 week after the last immunization (1 week before challenge). (D) Anti-Py blood stage IFA titers of mice eleven weeks post-boost (1 week before challenge). (E-H) The same data shown in Panels A-D are shown to provide a comparison between antibody titers at one and eleven weeks post-immunization. Black indicates responses at one week post-immunization and gray indicates responses at eleven weeks post-immunization. (E) Anti-Py sporozoite IFA titers elicited by immunization with a DNA-PyE140na prime and a HuAd5-PyE140na boost at one and eleven weeks post-boost. (F) Anti-Py sporozoite IFA titers elicited by immunization with a DNA-PyE140na prime and a HuAd5-PyE140co boost at one and eleven weeks post-boost. (G) Anti-Py blood stage IFA titers elicited by immunization with a DNA-PyE140na prime and a HuAd5-PyE140na boost at one and eleven weeks post-boost. (H) Anti-Py blood stage IFA titers elicited by immunization with a DNA-PyE140na prime and a HuAd5-PyE140co boost at one and eleven weeks post-boost. ****, ***, **, *, and NS indicate p<0.0001, p<0.001, p<0.01, p<0.05, and not significant by Mann-Whitney. (TIF) [file pone.0232234.s008.tif]

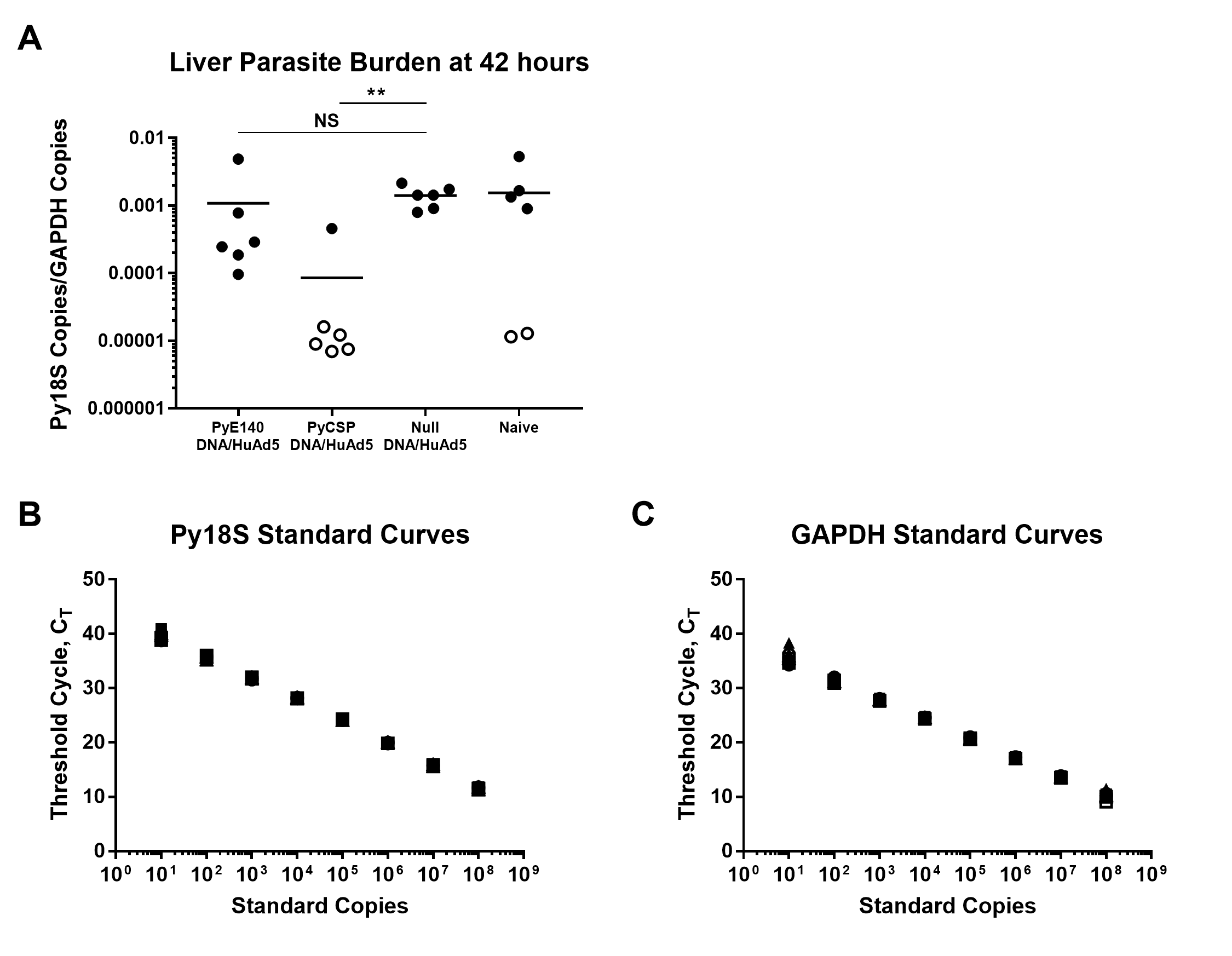

Supplement: S9 Fig — 11 week old CD1 mice were immunized with DNA vaccines encoding PyE140 or PyCSP at week 0 and HuAd5 vaccines encoding PyE140co or PyCSP at week 6. Groups of null-immunized and naïve mice were also included. Two weeks after the boost, mice were challenged with 5,000 P. yoelii 17XNL sporozoites by subcutaneous injection. Livers were harvested 42 hours after challenge for evaluation of Py18S mRNA copies/murine GAPDH mRNA copies by RT-qPCR. (A) Filled symbols represent detectable Py18S copies and open symbols represent Py18S copies below the limit of detection (10 copies of Py18S RNA) and are plotted using ½ the limit of detection (5 copies of Py18S mRNA). ** indicates p<0.01 and NS indicates not significant by Mann-Whitney. Standard curves for each run demonstrate reproducible quantification of (B) Py18S and (C) murine GAPDH mRNA. Data represent standard curves from eight plates run in replicates of two (n = 16 per standard dilution). Two outlier data points from the GAPDH standard curve were omitted from the total data analysis. Unique symbols identify data from each plate. (TIF) [file pone.0232234.s009.tif]

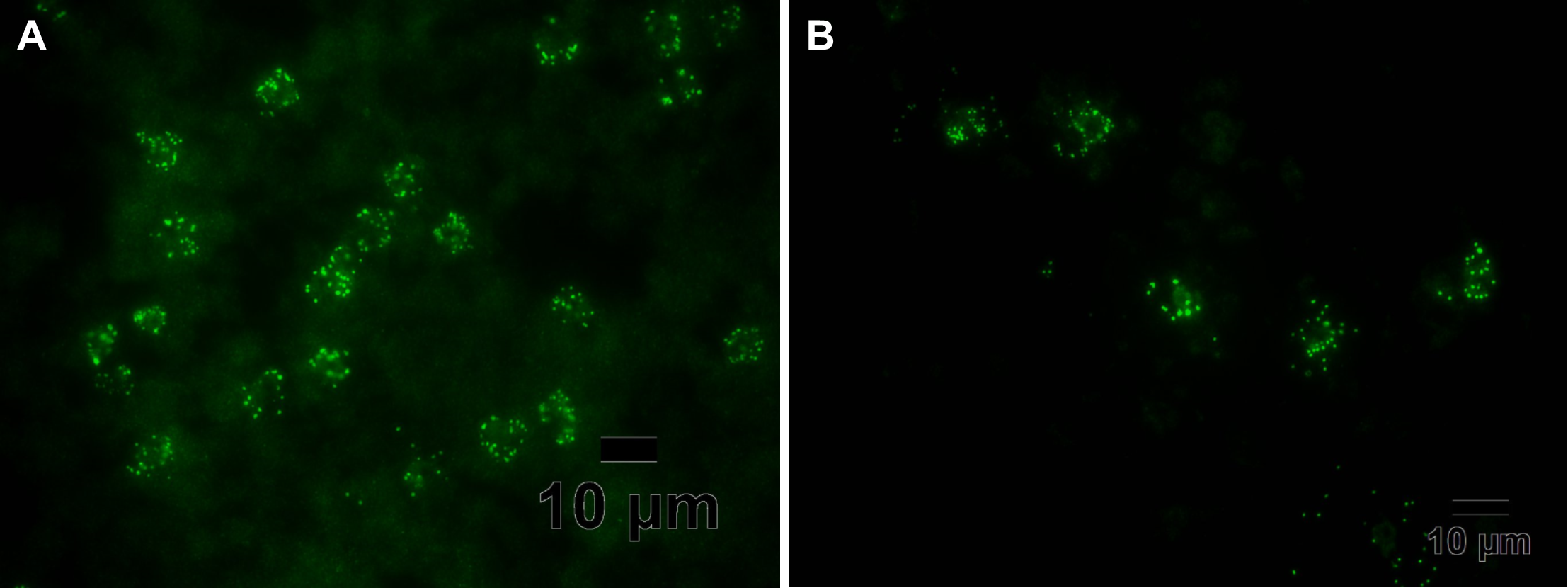

Supplement: S10 Fig — PfE140 expression in schizonts was detected by IFA. Sera from mice immunized against PfE140 were used to identify PfE140 expression in blood stage schizonts. Green staining indicates PfE140. PfE140 is expressed in individual merozoites in late stage (A) 3D7 strain and (B) 7G8 strain schizonts. Scale bar indicates 10 μm. (TIF) [file pone.0232234.s010.tif]
