# Supplementary material for: Novel malaria antigen Plasmodium yoelii E140 induces antibody-mediated sterile protection in mice against malaria challenge
Source: PLoS One. 2020 May 14;15(5):e0232234. doi: 10.1371/journal.pone.0232234 (PMC7224506; doi:10.1371/journal.pone.0232234)
Supplement: S1 Raw Images — (PDF) [file pone.0232234.s011.pdf]

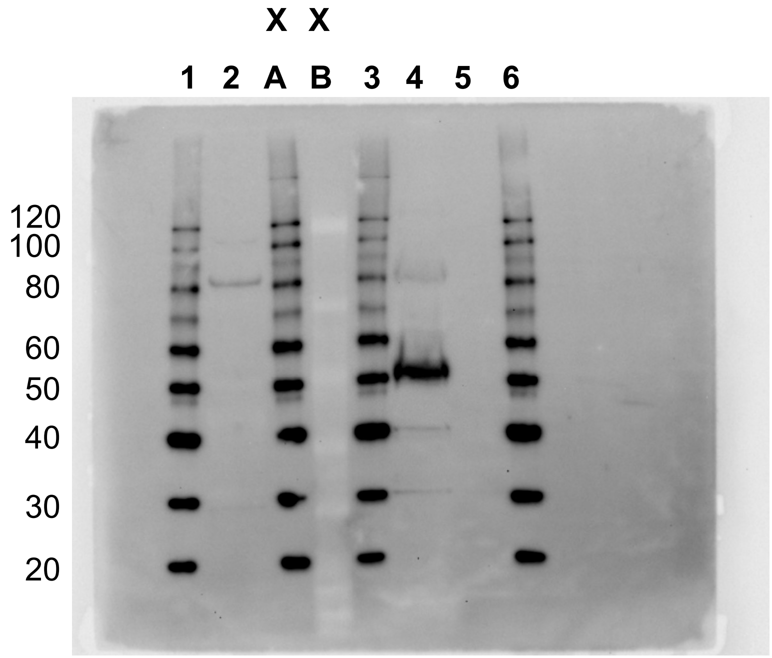

Fig 1B. Western blot analysis of *P. yoelii* salivary gland sporozoites (lane 2), mature schizonts (lane 4), and uninfected mouse red blood cells (lane 5). Parasite lysate was obtained from 3.6 × 10^5 *P. yoelii* sporozoites and 2 × 10^5 parasitized and non-infected red blood cells, respectively. Protein molecular weight standard (MagicMark XP, ThermoFisher Scientific, Inc.) is shown in lanes 1, **A**, 3, and 6 and the sizes in kDa are listed to the left for reference. **Lane B contains SeeBlue™ Plus2 Pre-stained Protein Standard (Invitrogen).** The blot was probed with polyclonal sera from mice immunized against PyE140 at a 1:500 dilution. The image was captured on a ChemiDoc Touch Imaging System (Bio-Rad Laboratories, Hercules, CA).

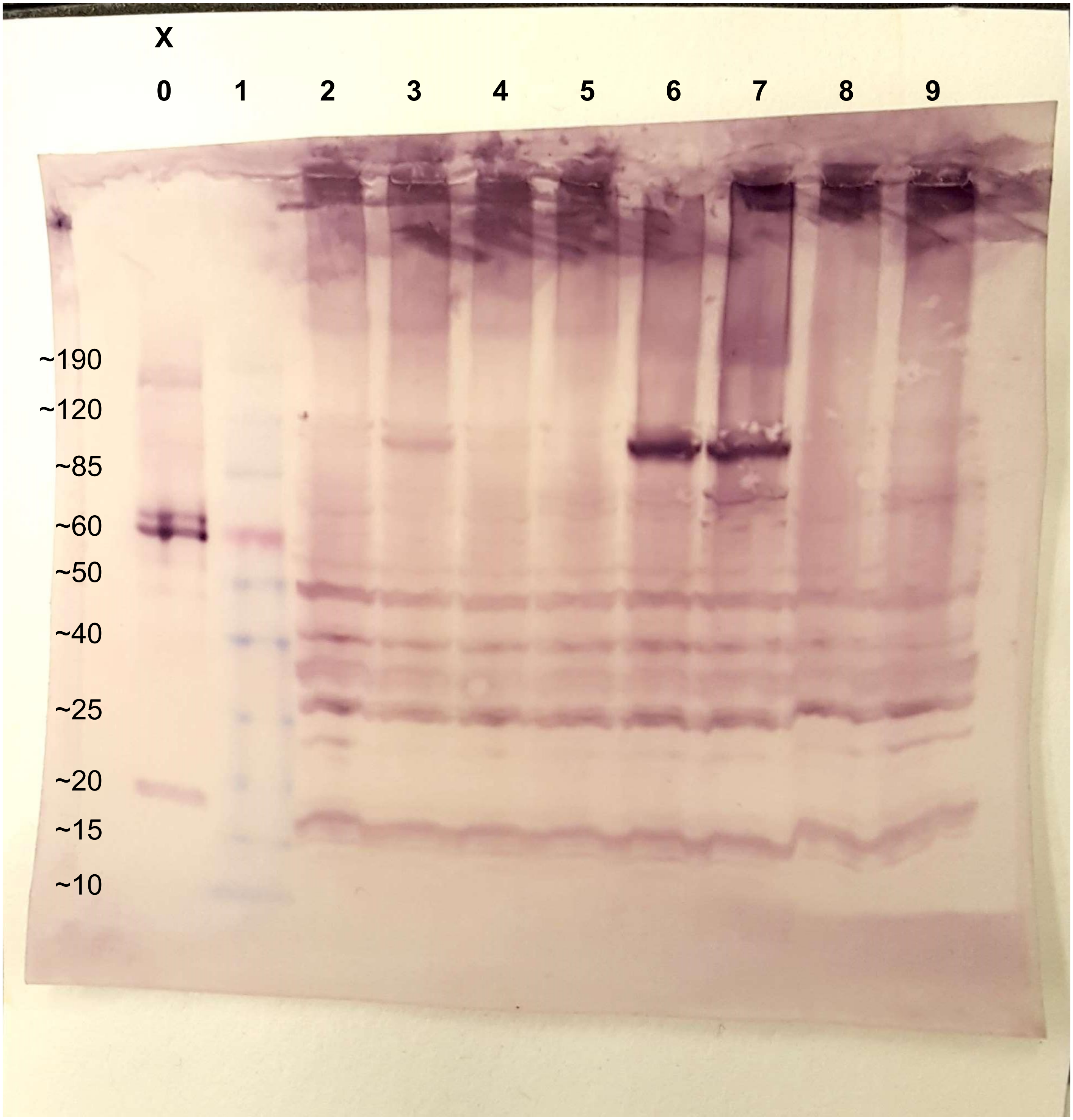

Fig S8A. 293-ORF6 cells were mock infected, transfected with 8 µg of DNA-PyE140na, infected with HuAd5 null, HuAd5-PyE140co or HuAd5-PyE140na at an MOI of 500 pu/cell and harvested 24 hours or 48 hours post-infection/transfection. **Lane 0, PyE140 C-terminal protein fragment expressed using the Wheat Germ Protein Expression System (CellFree Sciences Co., Ltd., Yokohama, Kanagawa, Japan),** Lane 1, Marker [BenchMark Prestained Protein Ladder (Invitrogen)]; Lane 2, Mock (48 hours); Lane 3, DNA-PyE140na (24 hours); Lane 4, HuAd5 null (24 hours); Lane 5, HuAd5 null (48 hours); Lane 6, HuAd5-PyE140co (24 hours); Lane 7, HuAd5-PyE140co (48 hours); Lane 8, HuAd5-PyE140na (24 hours), and Lane 9, HuAd5-PyE140na (48 hours). The primary antibody was sera from CD1 mice immunized with DNA and HuAd5 vectors expressing PyE140na and the secondary antibody was goat anti-mouse IgG conjugated to alkaline phosphatase. Signals were visualized with the KPL BCIP/NBT phosphate substrate system. The image was captured using an iPhone.

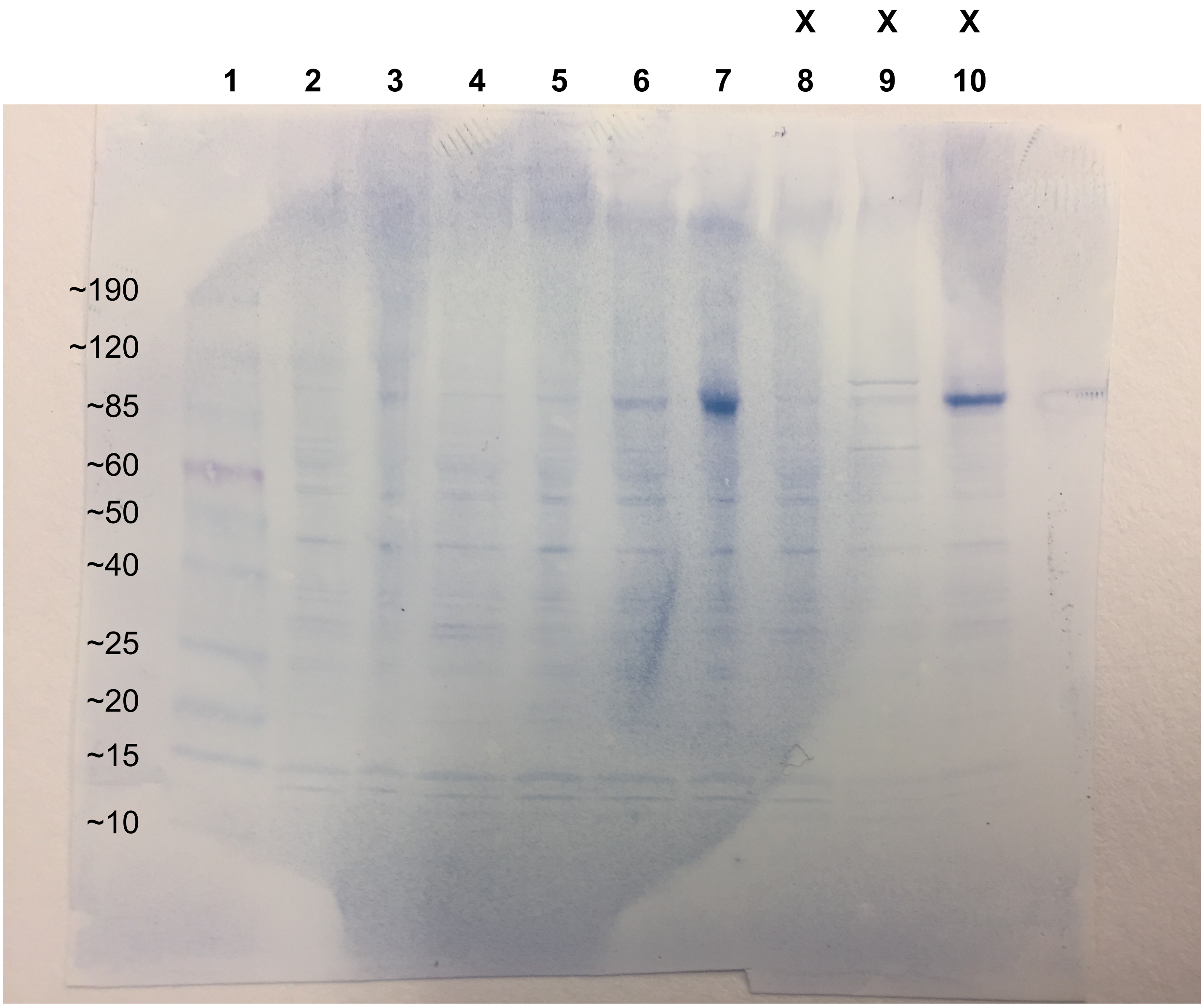

Fig S8B. 293-ORF6 cells were mock infected, transfected with 8 µg of DNA-PyE140na, infected with HuAd5 null, HuAd5-PyE140co or HuAd5-PyE140na and harvested 24 hours **or 48 hours** post-infection/transfection. Lane 1, Marker; Lane 2, Mock; Lane 3, DNA-PyE140na **24 hours**; Lane 4, HuAd5 null (MOI = 500) **24 hours**; Lane 5, HuAd5-PyE140na (MOI = 500) **24 hours**; Lane 6, HuAd5-PyE140na (MOI = 6,500) **24 hours**; Lane 7, HuAd5-PyE140co (MOI = 500) 24 hours; **Lane 8, HuAd5-PyE140na (MOI = 500) 48 hours; Lane 9, HuAd5-PyE140na (MOI = 6,500) 48 hours; Lane 10, HuAd5-PyE140co (MOI = 500) 48 hours**. The primary antibody was sera from CD1 mice immunized with DNA and HuAd5 vectors expressing PyE140na and the secondary antibody was goat anti-mouse IgG conjugated to alkaline phosphatase. Signals were visualized with the KPL BCIP/NBT phosphate substrate system. The image was captured using an iPhone.
